# Microdose Cocktail Study Reveals the Activity and Key Influencing Factors of OATP1B, P‐Gp, BCRP, and CYP3A in End‐Stage Renal Disease Patients

**DOI:** 10.1002/cpt.3546

**Published:** 2025-01-10

**Authors:** Weijie Kong, Yuejuan Pan, Yujie Wu, Yiyi Hu, Zhenbin Jiang, Xinkui Tian, Shuhong Bi, Song Wang, Feifei Feng, Yuyan Jin, Jiayu Li, Haiyan Li, Yue Wang, Hao Liang, Wen Tang, Dongyang Liu

**Affiliations:** ^1^ Department of Nephrology Peking University Third Hospital Beijing China; ^2^ Drug Clinical Trial Center Peking University Third Hospital Beijing China; ^3^ Institute of Medical Innovation Peking University Third Hospital Beijing China

## Abstract

OATP1B, P‐gp, BCRP, and CYP3A are the most contributing drug‐metabolizing enzymes or transporters (DMETs) for commonly prescribed medication. Their activities may change in end‐stage renal disease (ESRD) patients with large inter‐individual variabilities (IIVs), leading to altered substrate drug exposure and ultimately elevated safety risk. However, the changing extent and indictive influencing factors are not quantified so far. Here, a microdose cocktail regimen containing five sensitive substrate drugs (pitavastatin, dabigatran etexilate, rosuvastatin, midazolam, and atorvastatin) for these DMETs was administrated to Chinese healthy volunteers and ESRD patients. Drug pharmacokinetics profiles were determined, together with physiological, pharmacogenetic, and gut microbiome signature. Population pharmacokinetic and machine learning model were established to identify key influencing factors and quantify their contribution to drug exposure change. The exposure of pitavastatin, dabigatran, rosuvastatin, and atorvastatin increased to 1.8‐, 3.1‐, 1.1‐, and 1.3‐fold, respectively, whereas midazolam exposure decreased by 72% in ESRD patients. Notably, in addition to disease state, the relative abundance of genus *Veillonella* and *Clostridium_XIVb* were firstly identified as significant influencing factors for PTV and RSV apparent clearance, respectively, suggesting their indicative role for OATP and BCRP activity evaluation. Moreover, several genera were found to strongly associate with drug clearance and reduce unexplained IIVs. Accordingly, it was estimated that OATP1B and intestine P‐gp activity decreased by 35–75% and 29–44%, respectively, whereas BCRP and CYP3A4 activity may upregulate to some extent. Our study provides a quantitative and mechanistic understanding of individual DMET activity and could support precision medicine of substrate drugs in ESRD patients.

**Study Highlights**

**WHAT IS THE CURRENT KNOWLEDGE ON THE TOPIC?**

The activity of OATP1B, P‐gp, BCRP, and CYP3A may change in end‐stage renal disease (ESRD) patients with large inter‐individual variabilities (IIVs), leading to altered substrate drug exposure and ultimately elevated safety risk. However, the changing extent and indictive influencing factors are not quantified so far.

**WHAT QUESTION DID THIS STUDY ADDRESS?**

What is the changing extent, indictive influencing factors, and their respective contributions of OATP1B, P‐gp, BCRP, and CYP3A in ESRD patients?

**WHAT DOES THIS STUDY ADD TO OUR KNOWLEDGE?**

We found that the exposure of substrate drug pitavastatin, dabigatran, rosuvastatin, and atorvastatin increased to 1.8‐, 3.1‐, 1.1‐, and 1.3‐fold, respectively, whereas MDZ exposure decreased by 72% in ESRD patients. We identified several genera that strongly associate with drug clearance and reduce unexplained IIVs. Among them, genus *Veillonella* and *Clostridium_XIVb* were firstly identified as significant influencing factors for pitavastatin and rosuvastatin apparent clearance. It was estimated that OATP1B and intestine P‐gp activity decreased by 35–75% and 29–44%, respectively, whereas BCRP and CYP3A4 activity may upregulate to some extent in ESRD patients.

**HOW MIGHT THIS CHANGE CLINICAL PHARMACOLOGY OR TRANSLATIONAL SCIENCE?**

Our study provides a quantitative and mechanistic understanding of individual drug metabolizinig enzyme and transporter activity and could support precision medicine of substrate drugs in ESRD patients.

End‐stage renal disease (ESRD) is the most advanced stage of chronic kidney disease (CKD)^1^ with irreversible renal impairment and a declined life span.^2^ Drug exposure could significantly change due to altered renal and non‐renal elimination in CKD patients,^3^ leading to more common and severe safety issues,^4^ especially for ESRD patient who bears a heavy medication burden.^5^ Furthermore, highly unexplainable inter‐individual variabilities (IIVs) of drug exposure were observed in ESRD patients,^6^ further exacerbating their medication risk and bringing challenges for precision medicine.

The majority of drugs are eliminated through drug‐metabolizing enzymes and transporters (DMETs).^7^ OATP1B, P‐gp, BCRP, and CYP3A are among the most contributing DMETs for the absorption and disposition of newly approved drugs by the US Food and Drug Administration (FDA)^8^ and extensively prescribed drugs in the ESRD population.^5^ Although their activities in Caucasian and South Asian ESRD patients have been explored by the exposure of sensitive substrates obtained from clinical trials^9^, ^10^, ^11^ or systemic analysis,^12^, ^13^ they were not quantified in the Chinese population, one of the largest and fast‐growing ESRD population in the world.^14^ More importantly, the factors that are indicative for these DMET activities remain elusive and the source of IIV is therefore rarely explained.

Many demographic and physiological parameters were considered to be related to DMET function,^15^ including age,^16^ sex,^17^ parathyroid hormone (PTH) level,^18^ etc. Besides, gene polymorphisms are able to profoundly affect the PK profile of substrate drugs and contribute to the IIV of drug elimination.^19^ Uremic toxins were considered as one of the most important factors for the functional regulation of DMET in CKD whose influence could be partially reversed by dialysis.^20^ However, it is less indicative in ESRD patients since the extent of uremic toxins cleared by dialysis is variable.^21^ Instead, as one of the major sources of uremic toxin,^22^, ^23^ gut microbiome was demonstrated to significantly associate with P‐gp, BCRP, and CYP3A4 function by *in vivo* and *in vitro* studies.^24^, ^25^, ^26^ As a result, gut microbiome is a highly potential biomarker that could mechanically explain the change and IIVs of DMET function. Although many studies have been conducted to compare the gut microbiome signature between ESRD patients and healthy volunteers (HVs),^27^ the relationship between specific genera and DMET activities was rarely revealed and quantified.^28^ Taken together, the effect of ESRD on DMET activity is complex and may stem from multiple sources. Key influencing factors and their respective contributions to DMET activity in ESRD patients have not been dissected and quantified. As a result, predictive models for individual DMET activity that could explain IIVs have not been established and precision medicine by accurately predicting substrate pharmacokinetic (PK) profile in ESRD patients is still challenging.

Here, we conducted a microdose cocktail study in Chinese HVs and ESRD patients using pitavastatin (PTV), dabigatran etexilate (DABE), rosuvastatin (RSV), midazolam (MDZ), and atorvastatin (ATV) as sensitive substrate drugs to evaluate the activity alteration of OATP1B, P‐gp, BCRP, and CYP3A. Drug PK profiles and potential influencing factors were determined. Together, these data were analyzed using population pharmacokinetic (PPK) and machine learning (ML) models. We uncovered the quantitative change of these DMET activities, identified key indicative influencing factors, quantified their distributions to drug exposure and DMET activity, and established a prediction model for individual DMET activity in ESRD patients.

## METHODS

### Clinical study design

The study was designed as a single‐center, open‐label clinical trial to investigate the pharmacokinetics and tolerability of a microdose cocktail regimen (10 μg MDZ, 375 μg DABE, 10 μg PTV, 50 μg RSV, and 100 μg ATV) in Chinese HVs and ESRD patients. The study protocol was reviewed and approved by the Ethics Committee of Peking University Third Hospital, Beijing, China (No. M2021660). Written informed consents were obtained from all participants before the study was initiated. The trial was registered at clinicaltrials.gov (NCT05747768).

### Participants

We planned to enroll Chinese HVs and ESRD patients according to the following inclusion and exclusion criteria. Inclusion criteria included: (i) male or female; (ii) aged 18–65 years; (iii) body mass index (BMI) between 18.5 and 28.9 kg/m^2^; (iv) the estimated glomerular filtration rate (eGFR) calculated by the CKD‐EPI equation^29^ ≥90 mL/min/1.73 m^2^ (HV), or eGFR ≤15 mL/minute/1.73 m^2^ and regularly underwent dialysis three times a week (ESRD patient), respectively; and (v) non‐smokers or smokers with fewer than 20 cigarettes daily. ESRD patients with type 1 or type 2 diabetes mellitus, renal transplant, nephrectomy history, cancer, blood donation of 500 mL during 4 weeks prior to the study, unstable renal function (eGFR fluctuation >30% in the past 3 months), HIV, HCV, HBV, treponema pallidum or other severe diseases affecting OATP1B, P‐gp, BCRP, and CYP3A activities were excluded from this study. For HVs, additional exclusion criteria included hypoglycemia, glucose intolerance, and ketoacidosis. Moreover, no strong or moderate inhibitor and inducer drugs of these DMETs were concomitantly used in 14 days before and during the clinical trial.

### Drug dosing

The preparation of microdose cocktail regimen is described in **Methods** **S1**. Participants were fasted for at least 10 hour before administration with a single dose of microdose cocktail regimen. They were refrained to water and food for 2 and 4 hour after drug administration, respectively. To avoid the impact of dialysis, the regimen was taken 24 hour before dialysis.

### Pharmacokinetics assessment

Blood samples were collected at 0 hour (pre‐dose) and 0.5, 1, 2, 4, 8, 12, and 24 hour (post‐dose) for HVs, and at 0, 1, 4, 24 hour (before dialysis) and 28 hour (post‐dialysis) for ESRD patients. Each blood sample was divided to determine the concentration of MDZ, dabigatran (DAB, DABE major metabolite), and statins (including PTV, RSV, and ATV), respectively. In addition, the plasma protein binding of each drug was also determined. The bioanalysis and plasma protein binding process are described in **Methods** **S1**.

### Non‐compartmental analysis

The plasma concentration‐time curves were generated by R (version 4.3.3). All PK parameters were calculated by non‐compartmental analysis (NCA). Area under plasma concentration‐time curve up to 24 hour (AUC_last_) was calculated by the linear trapezoidal method for ascending and the log trapezoidal method for descending concentrations (linear up log down). Descriptive statistics were used to stratify the PK profiles with disease state (with or without ESRD), gender, and gene polymorphisms. NCA and descriptive statistics were conducted using Phoenix WinNolin (version 8.3.3).

### Gut microbiome

The stools of all ESRD patients and HVs devoid of antibiotics for at least 3 months were collected for 16S rRNA gene sequencing. The V3–V4 hypervariable regions of the bacteria 16S rRNA gene were amplified with primers 338F (5′‐ACT CCT ACG GGA GGC AGC AG‐3′) and 806R (5′‐GGA CTA CHV GGG TWT CTA AT‐3′) and sequenced using the MGI‐2000 platform (BGI, Shenzhen, China). Bioinformatics analysis was conducted to determine the relative abundance of each genus. The correlations between genera relative abundance and drug exposures (AUC_last_) were calculated by R (version 4.3.3).

### Pharmacogenetics

DNA was extracted from blood sample collected at 0 hour for each participant. Genotyping was conducted using matrix‐assisted laser desorption/ionization time‐of‐flight mass spectrometry (MALDI‐TOF MS) provided by MassARRAY from Agena Bioscience. The association between genotype and phenotype was defined according to the guidance from the Clinical Pharmacogenetics Implementation Consortium (https://www.pharmgkb.org/). For CYP3A5, normal, intermediate, and poor metabolizers were defined for rs776746 (CYP3A5 6986A>G). For OATP1B1 and BCRP, increased, normal, decreased, and poor function phenotypes were defined for rs2306283 (OATP1B1 388G>A), rs4149056 (OATP1B1 521T>C), and rs2231142 (BCRP 421C>A). No phenotypes were defined for other DMETs and single nucleotide polymorphisms (SNPs).

### PPK model

The time‐concentration data of both HVs and ESRD patients (before dialysis) were pooled together for each drug to establish its PPK model using nonlinear mixed‐effects modeling (NONMEM) software (version 7.2). Typical value and IIV were described using proper model structure. For each drug, a total of 167 variables were included to identify significant covariates, including demographic parameters, physiological parameters, gene polymorphisms, and the relative abundance of each gut microbiome genus. Disease state, gender, and gene polymorphism phenotype were treated as categorical variables. Goodness of fit (GOF), visual predictive check (VPC), and 500 steps bootstrap analysis were used to validate the model. The individual apparent clearance (CL/*F*) was estimated using the Bayes algorithm. All the modeling and simulation were carried out using the first‐order conditional estimation method with interaction (FOCE‐I) algorithm supported by PsN. Pirana was used for documentation of the development process.

### ML analysis

The correlation coefficient between the individual CL/*F* estimated by PPK models and the aforementioned variables was analyzed for each drug in ESRD patients. The variables significantly correlated with drug exposure (*P* < 0.05) were selected for multiple linear regression (MLR) analysis using the least absolute shrinkage and selection operator (LASSO) algorithm. The most predictive variables were selected by the minimum λ (λ min). The correlation coefficient analysis and LASSO were performed with R (version 4.1.3).

### Simulation

Simulations were conducted for each drug in each participant using the established PPK model. Dosage was set according to the microdose cocktail regimen. The entire concentration‐time curves were generated using predicted individual data from 0 hour (pre‐dose) to 150 hour (post‐dose). All PK parameters were calculated using NCA.

## RESULTS

### Demographic

The workflow of the study is illustrated in **Figure** **S1**. A total of 14 HVs and 10 ESRD patients under dialysis were enrolled in this study. All participants finished the clinical trial without other treatment. The demographic data, complications, and concomitant medication are shown in **Table** **1**. Age and BMI were similar between the HV and ESRD groups. Significantly decreased total bilirubin, hematocrit, hemoglobin, and serum albumin were observed in ESRD patients. All ESRD patients suffered from multiple complications and therefore bear heavy concomitant medications.

**Table 1:** Demographic data of the HV and ESRD groups

Parameter	HV group	ESRD group	*P* value
*N*	14	10	
Male/female (*n*/*n*)	5/9	7/3	
Age (year)	35.6 (36%)^a^	43.4 (12%)	0.09
BMI (kg/m^2^)	22.4 (11%)	21.9 (13%)	0.69
Serum creatinine (μmol/L)	70.1 (14%)	1,150.3 (23%)	<0.05
eGFR (mL/minute/1.73 m^2^)	106.6 (8%)	4 (19%)	<0.05
Total protein (g/L)	71.9 (4%)	72.2 (4%)	0.79
Serum albumin (g/L)	44.9 (8%)	42.1 (4%)	<0.05
Serum globulin (g/L)	27.2 (12%)	30.1 (9%)	<0.05
Alanine aminotransferase (U/L)	13.1 (40%)	16.8 (142%)	0.59
Aspartate aminotransferase (U/L)	18.2 (27%)	14.5 (74%)	0.29
Total bilirubin (μmol/L)	14.9 (51%)	6.4 (20%)	<0.05
Alkaline phosphatase (U/L)	67.1 (28%)	82.8 (38%)	0.16
Hematocrit	0.44 (20%)	0.35 (9%)	<0.05
Hemoglobin (g/L)	137.1 (12%)	112.9 (8%)	<0.05
Complications			
Hypertension	NA	50%	
Anemia	NA	100%	
Hyperphosphatemia	NA	100%	
Hyperkalemia	NA	30%	
Secondary hyperparathyroidism	NA	70%	
Chronic glomerulonephritis	NA	100%	
Concomitant medications			
Antihypertension	NA	50%	
Antianemia	NA	100%	
Antithrombotic	NA	90%	
Phosphate binding	NA	90%	
Calcitriol and vitamin D analogs	NA	70%	

^a^ Parameters are displayed as mean (CV%).

### Safety

All participants finished the study, without treatment‐emergent adverse events (AEs), serious AEs, deaths, or treatment‐emergent AEs leading to discontinuation of the study drug, suggesting that the regimen is well tolerated in both Chinese HVs and ESRD patients.

### Bioanalysis

The linearity, accuracy, precision, tested stabilities, and recoveries of MDZ, DAB, and statins for all three batch validation assays and the linearity, accuracy, and precision for bioassay batch met the guidance from the US Food and Drug Administration and Chinese NMPA (**Tables** **S1** **and** **S2**) (https://www.fda.gov/regulatory‐information/search‐fda‐guidance‐documents/m10‐bioanalytical‐method‐validation; https://ydz.chp.org.cn/#/item?bookId=4&entryId=5692).

In total, drug concentrations of 162 plasma samples were determined. Samples from one ESRD patient were excluded from ATV determination since she took ATV for treatment within 14 days before the study. The quantifications of nine MDZ, one DAB, and six statin samples failed due to inadequate plasma samples. The unbound fraction (*f*_u_) was measured using plasma samples before dosing in this trial and the results were listed in **Table** **S3**. The *f*_u_ of all high protein‐binding drugs (MDZ, PTV, RSV, and ATV) increased to varying extent.

### Pharmacokinetic profiles

**Figure 1:**
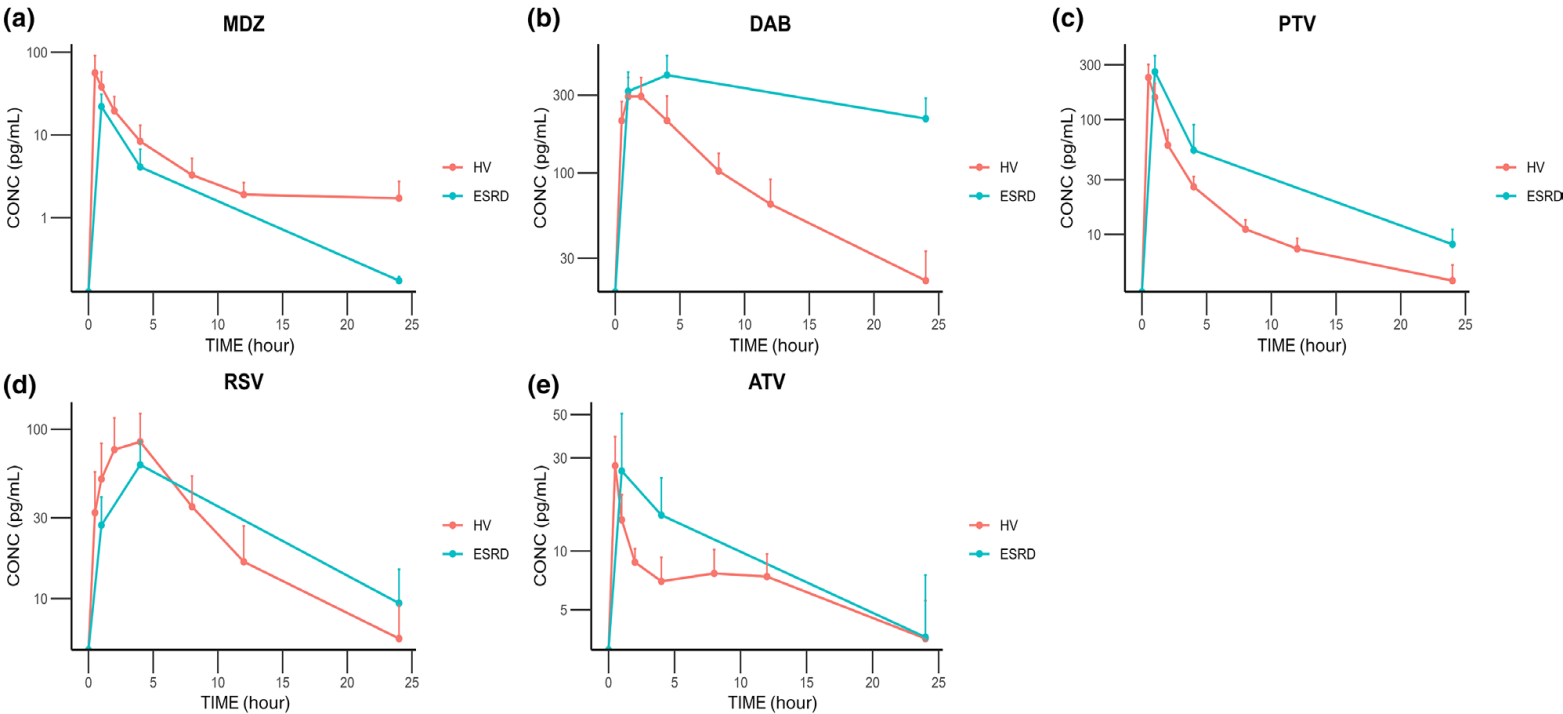
Concentration‐time curve of MDZ (**a**), DAB (**b**), PTV (**c**), RSV (**d**), and ATV (**e**). The data of HV and ESRD are displayed as red and blue lines, respectively.

The concentration‐time curves and exposure parameters (*C*_max_ and AUC_last_) of all drugs are shown in **Figure** **1** and **Table** **2**. Except for MDZ whose AUC_last_ decreased by 72%, the exposures of all drugs increased to some extent in ESRD patients. The AUC_last_ of PTV and DAB in ESRD patients were 1.8‐ and 3.1‐fold of that in HV (*P* < 0.05). On the contrary, similar exposures were observed for RSV (1.1‐fold) and ATV (1.3‐fold) between the two groups. Gender (**Figure** **S2**), age (**Figure** **S3**), and PTH (**Figure** **S4**) had no significant impact on all drug exposure.

**Table 2:** Calculated PK parameters of each drug in two groups

Drug	Group	HV	ESRD	ESRD/HV ratio	*P* value	ESRD/HV ratio (Caucasian)^a^
PTV	*C*_max_ (pg/mL)	222 (183–270)^b^	246 (190–319)	1.11 (0.82, 1.49)	0.42	1.47 (0.91, 2.38)
	AUC_last_ (hour·pg/mL)	500 (439–569)	922 (686–1,239)	1.84 (1.41, 2.42)	<0.05	1.30 (0.74, 2.27)
DAB	*C*_max_ (pg/mL)	303 (260–354)	384 (317–467)	1.27 (1.01–1.60)	0.06	0.72 (0.38, 1.38)
	AUC_last_ (hour·pg/mL)	2,233 (1,896–2,631)	6,872 (5,573–8,473)	3.08 (2.40, 3.94)	<0.05	3.39 (1.78, 6.44)
RSV	*C*_max_ (pg/mL)	73.0 (52.6–101)	58.6 (46.5–73.9)	0.80 (0.53, 1.22)	0.16	1.04 (0.45, 2.41)
	AUC_last_ (hour·pg/mL)	591 (440–794)	656 (513–840)	1.11 (0.75, 1.63)	0.80	0.71 (0.27, 1.86)
MDZ	*C*_max_ (pg/mL)	48.8 (35.0–67.9)	16.6 (9.62–28.7)	0.34 (0.19, −0.60)	<0.05	0.55 (0.36, 0.83)
	AUC_last_ (hour·pg/mL)	121 (91.7–160)	34.0 (20.9–55.4)	0.28 (0.17, 0.46)	<0.05	0.40 (0.23, 0.70)
ATV	*C*_max_ (pg/mL)	23.6 (17.5–32.0)	22.0 (13.0–37.1)	0.84 (0.50, 1,39)	0.85	2.94 (1.28, 6.77)
	AUC_last_ (hour·pg/mL)	141 (111–178)	181 (112–292)	1.26 (0.79, 2.05)	0.10	1.13 (0.57, 2.22)

^a^ The data was obtained from literature.^10^

^b^ The parameter is shown as geometry mean (95% CI).

The unbound concentration‐time curves for each drug in HVs and ESRD are displayed in **Figure** **S5**, along with the corresponding exposure parameters (**Table** **S4**). Notably, the decrease in magnitude of unbound MDZ AUC_last_ in ESRD patients was reduced compared with that of total MDZ AUC_last_ (54% vs. 72%). In addition, the unbound exposures of RSV (2.9‐fold) and ATV (2.0‐fold) were significantly larger in ESRD patients (*P* < 0.05).

### Gene polymorphisms

To explore the effect of gene polymorphisms on DMET function, 19 SNPs were determined (**Table** **S5**). Genotypes have no significant effect on drug exposure (**Figures** **S6** **and** **S7**). Moreover, drug exposure was also stratified by OATP1B1 (rs2306283 and rs4149056), BCRP (rs2231142), and CYP3A5 (rs776746) phenotypes. Though not significant, an increasing trend was observed for PTV exposure across OATP1B1 increased, normal, decreased, and poor function phenotype in ESRD patients, while larger RSV exposure was observed in BCRP poor function phenotype in both HVs and ESRD patients (**Figures** **S8** **and** **S9**).

### Gut microbiome

**Figure 2:**
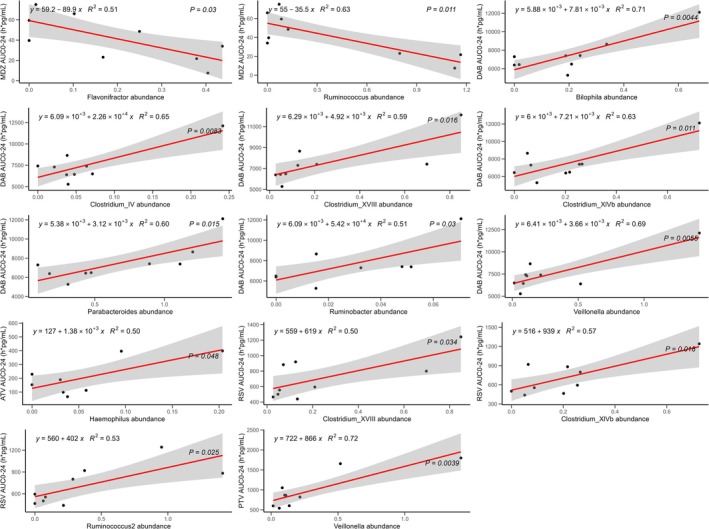
Correlations between drug exposure and specific bacterial genera in ESRD patients.

Gut microbiome of nine ESRD patients and nine HVs that met the requirement were compared. Though no statistical difference was observed in both α‐ (**Figure** **S10A**,**B**) and β‐diversity (**Figure** **S10C**), the relative abundance of specific genera was differentially regulated in HVs and ESRD patients (**Figure** **S10D**). The ESRD‐elevated representatives include *Facecalibacterium*, *Phascolarctobacterium*, *Roseburia*, and *Blautia*, while the ESRD‐depleted representatives include *Prevotella* and *Megamonas* (**Figure** **S10E**). Correlations between the relative abundance of each genus and drug exposure results were analyzed and those with significance (*p* < 0.05) were regarded as potential influencing factors indicative for DMET activities (**Figure** **2**).

### PPK model

Two‐compartmental models with first‐order absorption and first‐order elimination were used to capture the PK characteristic of all five drugs. The absorption rate constant (*k*_a_), CL/*F*, volume of distribution of the central (*V*_c_/*F*) and peripheral (*V*_p_/*F*) compartments, and inter‐compartment clearance (*Q*/*F*) were estimated (**Table** **3**). Model reliability was fully assessed by GOF, VPC (**Figures** **S11** **–** **S20**), and bootstrap analysis (**Table** **3**). The effect of disease state (defined as with or without ESRD) on CL/*F* was identified as the significant covariate for PTV, DAB, and MDZ (Eqs. (1), (2), (3)). Notably, the relative abundance of genus *Veillonella* and *Clostridium_XIVb* was also found to significantly affect CL/*F* of PTV and RSV, respectively (Eqs. 1 and 4).

(1) $$\mathrm{CL}/F\;\left(\mathrm{PTV}\right)=17\times e^{0.096}\times {\left(\frac{e^{\text{Veil}}}{1.08}\right)}^{-0.68}\times \left(1+{\mathrm{DIS}}_{\mathrm{PTV}}\right)$$

(2) $$\mathrm{CL}/F\;\left(\mathrm{DAB}\right)=146\times e^{0.225}\times \left(1+{\mathrm{DIS}}_{\mathrm{DAB}}\right)$$

(3) $$\mathrm{CL}/F\left(\mathrm{MDZ}\right)=68\times e^{0.379}\times \left(1+{\mathrm{DIS}}_{\mathrm{MDZ}}\right)$$

(4) $$\mathrm{CL}/F\;\left(\mathrm{RSV}\right)=43\times e^{0.508}\times {\left(\frac{e^{\text{Clos}}}{1.08}\right)}^{-3.3}$$

(5) $$\mathrm{CL}/F\;\left(\mathrm{ATV}\right)=363\times e^{0.48}$$

${\mathrm{DIS}}_{\mathrm{PTV}}$, ${\mathrm{DIS}}_{\mathrm{DAB}}$, and ${\mathrm{DIS}}_{\mathrm{MDZ}}$ were the effect of disease state on drug CL/*F* of PTV, DAB, and MDZ, respectively, and their values are displayed in **Table** **3**. Veil and Clos are the relative abundance of *Veillonella* and *Clostridium_XIVb*, respectively.

**Table 3:** Estimated PPK final model parameters with 500 bootstraps

Parameter	Unit	Estimate (RSE%)	IIV% (RSE%)	Bootstrap median (90% CI)
PTV				
*k*_a_	1/hour	18 (NA)	NA	18 (18, 18)
CL/*F*	L/hour	16.5 (4.50)	9.60 (37.6)	17 (15, 18)
DIS_PTV_ on CL/*F*		−0.381 (10.8)	NA	−0.37 (−0.47, −0.29)
Veil on CL/*F*		−0.677 (21.7)	NA	−0.79 (−1.2, −0.14)
*V*_c_/*F*	L	25.2 (7.00)	NA	25 (22, 29)
*V*_p_/*F*	L	76.1 (9.90)	NA	78 (65, 87)
*Q*/*F*	L/hour	8.01 (0)	7.60	8.1 (6.6, 9.4)
DAB				
*k*_a_	1/hour	1.22 (19.8)	21.5 (56.0)	1.0 (0.51, 1.93)
CL/*F*	L/hour	146 (12.5)	22.5 (24.3)	145 (128, 165)
DIS_DZB_ on CL/*F*		−0.869 (6.40)	NA	−0.86 (−0.91, −0.82)
*V*_c_/*F*	L	794 (17.3)	25.9 (29.0)	656 (372, 1,215)
*V*_p_/*F*	L	429 (58.3)	86.0 (40.9)	528 (129, 728)
*Q*/*F*	L/hour	92.5 (30.9)	NA	107 (32, 153)
RSV				
*k*_a_	1/hour	0.30 (17.4)	NA	0.30 (0.164,0.352)
CL/*F*	L/hour	43.1 (8.60)	50.8 (30.4)	48 (29, 57)
Clos on CL/*F*		−3.26 (54.3)	NA	−2.9 (−4.8, −1.7)
*V*_c_/*F*	L	289 (17.2)	22.4 (59.4)	282 (197, 382)
ALB on *V*_c_/*F*		−6.42 (24.3)	NA	−6.6 (−8.0, −4.9)
*V*_p_/*F*	L	2,740 (22.5)	NA	2,407 (1,893, 3,589)
*Q*/*F*	L/hour	48.6 (11.7)	NA	45 (35, 62)
MDZ				
*k*_a_	1/hour	13 (NA)	NA	13 (13, 13)
CL/*F*	L/hour	67.6 (24.6)	37.9 (27.3)	68 (52, 84)
DIS_MDZ_ on CL/*F*		0.94 (52.1)	NA	0.96 (0.20, 1.68)
*V*_c_/*F*	L	195 (18.8)	42.1 (43.2)	188 (137, 252)
*V*_p_/*F*	L	333 (25.6)	NA	358 (86, 580)
*Q*/*F*	L/hour	34.7 (15.5)	NA	36 (24, 45)
ATV				
*k*_a_	1/hour	7.53 (16.7)	NA	22 (−36, 57)
CL/*F*	L/hour	363 (9.20)	48.0 (44.6)	356 (280, 458)
*V*_c_/*F*	L	835 (37.2)	99.3 (63.3)	821 (226, 1,930)
*V*_p_/*F*	L	5,830 (2.10)	62.6 (19.1)	5,971 (4,228, 7,339)
*Q*/*F*	L/hour	3,920 (3.80)	30.2 (84.7)	3,302 (2,185, 5,013)

ALB, serum albumin concentration; CL/*F*, apparent clearance; Clos, genus *Clostridium_XlVb* abundance; DIS, disease state (with or without ESRD); *k*_a_, first‐order absorption rate constant; *Q*/*F*, Apparent clearance between *V*_c_/*F* and *V*_p_/*F*; *V*_c_/*F*, Apparent volume of distribution for the central compartment; *V*_p_/*F*, Apparent volume of distribution for the peripheral compartment; Veil, genus *Veillonella* abundance.

### DMET activity change

As the sensitive substrate, the exposure change of PTV is indictive for OAPT1B function. As suggested by the PPK model, OATP1B activity reduction originates from the direct effect of ESRD (−0.38) and an additional effect from the relative abundance of the genus *Veillonella* (−0.677). Taken together, we estimated that the PTV activity could decrease by 37–75% in ESRD patients (Eq. 1).

As the prodrug DABE, but not DAB, is exported by intestine P‐gp, the exposure of DAB is specifically indictive for the function of intestine P‐gp. DAB is predominately eliminated through glomerular filtration (80–85%). PPK model demonstrated that ESRD decreased CL/*F* by an estimate of around 87% (Eq. 2). Collectively, the function of intestine P‐gp could decrease by 29–44%, as suggested by Eqs. 6 and 7.

(6) $$\frac{{\mathrm{CL}}_{\mathrm{DAB},\text{ESRD}}}{F_{\mathrm{DAB},\text{ESRD}}}=\left(1-{\text{effect}}_{\text{ESRD}}\right)\times \frac{{\mathrm{CL}}_{\mathrm{DAB},\mathrm{HV}}}{F_{\mathrm{DAB},\mathrm{HV}}}$$

(7) $$\frac{{\mathrm{CL}}_{\mathrm{DAB},\text{ESRD}}}{{\mathrm{CL}}_{\mathrm{DAB},\mathrm{HV}}}=R_{\mathrm{nf}}+R_{f}\times \frac{{\text{eGFR}}_{\text{ESRD}}}{{\text{eGFR}}_{\mathrm{HV}}}$$

${\mathrm{CL}}_{\mathrm{DAB},\text{ESRD}}$, ${\mathrm{CL}}_{\mathrm{DAB},\mathrm{HV}}$, $F_{\mathrm{DAB},\text{ESRD}}$, and $F_{\mathrm{DAB},\mathrm{HV}}$ are the clearance and bioavailability of DAB in ESRD patients and HVs, respectively. ${\text{effect}}_{\text{ESRD}}$ is the effect of ESRD on CL/*F* of DAB. ${\text{eGFR}}_{\text{ESRD}}$ and ${\text{eGFR}}_{\mathrm{HV}}$ are the mean eGFR in ESRD patients and HVs, respectively. $R_{\mathrm{nf}}$ and $R_{f}$ are the partition of DAB elimination by non‐filtration (15–20%) and filtration route (80–85%).

For RSV and ATV, the effect of ESRD on CL/*F* was not determined, consistent with their indistinguishable exposure between groups. As RSV is a substrate of both OATP1B and BCRP, the smaller increase in magnitude of drug exposure (11%) suggested that BCRP function might be upregulated in ESRD patients that compensate for the functional loss of OATP1B, though the exact magnitude could not be estimated.

At last, ESRD exerts a positive effect on MDZ CL/*F* with an estimation of 0.94. However, we cannot conclude the same magnitude of CYP3A activity augment, since the fraction of unbound MDZ significantly increased in ESRD patients and may partially explain the increase in drug clearance.^10^

### ML analysis

MLR models were established for the CL/*F* of all drugs in ESRD patients. LASSO coefficient profiles and tuning parameter selection are displayed in **Figures** **S21** **and** **S22**. The included variables, together with corresponding regression coefficients and intercepts, are listed in **Table** **S6** and Eqs. (8), (9), (10), (11), (12). Apart from the relative abundance of Veillonella, additional physiological parameters and gut microbiome genera abundance were suggested by ML analysis.

$$\mathrm{CL}/F\;\left(\mathrm{PTV}\right)=0.0124\times \text{Phascolarctobacterium}-0.273\times \text{Urea}$$

(8) −2.20×Veillonella+17.5

(9) $$\begin{array}{c} \mathrm{CL}/F\;\left(\mathrm{DAB}\right)=-46.4\times \text{Hematocrit}-8.77\\ \;\times \text{Clostridium}\_\text{XVIII}+44.1 \end{array}$$

(10) $$\mathrm{CL}/F\;\left(\mathrm{RSV}\right)=1.51\times \mathrm{Age}+5.16\times \text{creatine kinase}\_\mathrm{MB}-57.9$$

(11) $$\begin{array}{c} \mathrm{CL}/F\;\left(\mathrm{MDZ}\right)=11.6\times \text{Triglyceride}-17.7\times \text{Anaerostipes}\\ \;+0.259\times \text{Platelet count}+57.4 \end{array}$$

(12) $$\mathrm{CL}/F\;\left(\mathrm{ATV}\right)=8.92\times \text{Height}+18.6\times \text{Sutterella}-1152$$

Triglyceride is plasma triglyceride concentration, while Anaerostipes, Clostridium_XVIII, Phascolarctobacterium, Veillonella, and Sutterella represent their respective relative genera abundance.

### Simulation

The calculated PK parameters for the simulated PK profiles are listed in **Table** **S7**. Since PPK models were constructed using PK data from HVs and ESRD patients before dialysis, the simulated concentration‐time was assumed to be devoid of dialysis influence. Thus, the comparison of predicted and measured concentration at 28 hour could be used to evaluate the effect of dialysis on drug elimination. As shown in **Table** **S8**, dialysis could profoundly remove DAB but has limited capability on the elimination of other drugs that are high protein binding.

## DISCUSSION

In this study, we administrated a microdose cocktail regimen containing five sensitive substrate drugs for four key DMETs to Chinese HVs and ESRD patients. With the aid of PPK and ML analysis, we identified specific gut microbiome genera and physiological parameters that significantly associated their PK profiles and explained their IIVs. Accordingly, we developed predictive models to assess their individual drug exposure in ESRD patients. In addition, the change in respective DMET activity was also estimated using substrate drug exposure.

According to PTV PPK model, the direct effect of ESRD on OATP1B activity was −38%. The magnitude is lower than the previous report that estimated a 60% reduction in OATP1B in severe CKD patients.^30^ The reduction was entirely ascribed to the disease state without consideration of genus *Veillonella* relative abundance, which ranged from 0.015 to 1.42 in our study. When taking *Veillonella* relative abundance into consideration, OATP1B activity could decrease up to 75% in ESRD patients with the largest relative abundance. For other patients whose *Veillonella* relative abundance ranged from 0.015% to 0.52%, the impairment of OATP1B activity was mild with a mean reduction of around 41% (35–54%). The magnitude was also verified by coproporphyrin I (CP‐I), an OATP1B‐specific endogenous biomarker. CP‐I level increased by 2.5‐fold in Caucasian ESRD patients compared to HVs.^10^ Given the 15% fraction of urine excretion of CP‐I, which is totally lost in ESRD patients, the calculated remaining OATP1B1 function is 47%. More importantly, the inclusion of disease state and genus *Veillonella* relative abundance could markedly decrease the IIV of PTV (34.8–9.6%), more profound than well‐known gene polymorphism phenotypes (34.8–31.3%). Except for *Veillonella*, LASSO also identified additional influencing factors that are absent in the PPK model, including genus *Phascolarctobacterium* relative abundance (ranged between 0.005% and 37%, accounting for around 5% of *Veillonella* contribution) and plasma urea concentration (an indicator of disease state). Notably, the effect of OATP1B activity alteration varies depending on the test statins, even though they share OATP1B for hepatic uptake. We reckoned that the reason for the discrepancy lies in the different role that OATP1B plays in statin elimination, as demonstrated by the varying effect of the *SLCO1B1* genotype on statin exposure.^31^

DABE was rapidly and fully converted into DAB after absorption by carboxylesterase in HVs. Although carboxylesterase activity was elevated in ESRD patients,^32^ it has an ignorable effect on DAB exposure. As a result, the change in DAB exposure should be ascribed to P‐gp activity alteration. P‐gp function impairment in Chinese ESRD patients was estimated at 29–44%, which is comparable to that in severe Caucasian CKD patients (40%).^10^ After the inclusion of disease state, the IIV of CL/*F* dramatically reduced (95.7–22.5%). The relative abundance of genus *Bilophila* and *Clostridium_IV*, though not included in the final model, were strongly correlated with DAB exposure (*p* < 0.05) with *r* ^2^ of 0.71 and 0.65, respectively, and contributed to the reduction of CL/*F* IIV to a less extent. Of note, *Bilophila* abundance was also associated with the exposure of tacrolimus (P‐gp substrate).^33^ Genus *Clostridium_XVIII* abundance was also identified by MLR analysis. However, their association with P‐gp activity needs to be further clarified in the future.

Addressing BCRP activity is challengeable due to lack of specific substrate. RSV, whose CL/*F* decreased by 9%, is eliminated by both efflux transporter BCRP and uptake transporter OATP1B. Given the 38% reduction in OATP1B activity, BCRP activity was postulated to be upregulated, although the exact magnitude was hard to quantify. Consistently, ileum BCRP expression increased by around 100% in CKD rat.^34^ Furthermore, we identified genus *Clostridium_XlVb* relative abundance as a significant covariate on CL/*F*, although the reduction in IIV is limited (55.4–50.8%). In addition, the inclusion of serum albumin concentration, an indicator of disease state, markedly reduces *V*_c_/*F* IIV (56.3–22.4%).

Similarly, upregulated activity was observed in CYP3A as suggested by the decreased exposure of MDZ. However, given the increased *f*_u_ and unbound clearance of MDZ, the extent of activity alteration of CYP3A is still unknown. Besides, the exposure of ATV was the combinatorial effect of functional change of CYP3A4, OATP1B, P‐gp, and BCRP. Thus, the decrease in ATV exposure attributable to upregulation of CYP3A activity may be compensated by the functional impairment of OATP1B. The measurement of MDZ and ATV metabolite is helpful for better evaluation of CYP3A activity. However, the plasma volume was not sufficient for the analysis of both parent drugs and their corresponding metabolites under microdose. Further investigation is warranted in the future.

The magnitude of exposure alteration that ESRD exerts was compared between the Chinese and Caucasian population.^10^ The AUC ratio of PTV between ESRD patients and HVs (AUCR_ESRD/HV_) in the Chinese population is 42% higher than that in the Caucasian population (1.84 vs. 1.30). Similar trends were also observed for RSV (1.11 vs. 0.71) and ATV (1.26 vs. 1.13), although the magnitudes varied. On the contrary, DAB exposure increment was consistently observed in the Chinese (3.08) and Caucasian population (3.39). As for MDZ, a larger reduction was also found in the Chinese population (0.28 vs. 0.40). However, we could not conclude that there are obvious inter‐ethnic differences in the effect of ESRD on DMET activity, due to the limited sample size and lack of influencing factor information in the Caucasian population. Further studies are warranted in the future.

It was estimated that 50% of CKD patients are taking statins for the treatment of atherosclerotic cardiovascular disease associated with the impairment of renal function.^35^, ^36^ PTV, RSV, and ATV are all among the most commonly prescribed statins in the CKD population.^37^ The exposure to statins increases to varying extent in ESRD patients^10^ and associates with elevated rhabdomyolysis risk.^38^ In addition, ESRD increases thromboembolic risk among patients with atrial fibrillation,^39^ which is found to be associated with poor outcomes in ESRD population.^40^, ^41^ Though prohibited, the off‐label use of DABE in ESRD patients widely occurs in routine clinical practice and may be associated with adverse outcomes.^42^ At last, the use of MDZ is associated with prolonged sedation prolonged sedation due to accumulation of conjugated metabolites in severe CKD patients.^43^ Taken together, our study could support the precise use of these substrate drugs.

PPK model is widely accepted to for the identification of key influencing factors significantly impactful on PK parameters.^44^ MLR, another powerful tool,^45^ provided an alternative perspective that is complementary to the PPK model. On one hand, the conduction of MLR analysis relied on the individual CL/*F* predicted by the PPK model. On the other hand, MLR successfully identified several influencing factors that are absent in the PPK model and thereby exhibited the potential to further decrease the unexplained IIVs. Collectively, our study may present a novel strategy for the construction of a predictive model by the combination of pharmacometrics and ML methods.

There are several limitations in our study. The sample size is relatively limited, especially for ESRD patients. The well‐known confounding factors, such as age and BMI, were matched between ESRD patients and HVs to avoid bias. In addition, drug concentrations from both ESRD patients and HVs were pooled together and subjected to PPK analysis to maximize the use of all available data. Another limitation is the projection of drug exposure to DMET activity. The drugs we used in this study are sensitive to the activity alteration of their respective DMET. However, the impact of *f*_u_ was not considered, which may introduce bias in the evaluation of DMET activity. It was reported that MDZ exposure was affected by both CYP3A4 activity and *f*_u_ ^20^ and free drug clearance of MDZ was not different between ESRD and HVs after intravenous administration.^46^ Consistently, *f*_u_ increased by 62% in our study, which could partially explain the decreased MDZ exposure. Thus, the magnitude of CYP3A function may be overestimated. On the contrary, *f*_u_ increase of PTV, RSV, and ATV may lead to underestimation of respective DMET activity impairment. DMET activity will be further evaluated in the future using physiologically based pharmacokinetic models.

In summary, we conducted a clinical study using a microdose cocktail regimen to probe the functional change of multiple DMET in Chinese ESRD patients. Key influencing factors from demographic, physiological, gene polymorphisms, and gut microbiome parameters were identified and their contributions to individual drug exposure were quantified. Although the sample size is limited to due enrollment difficulties, we believe our study could provide insight into the precision medicine of CKD patients through comprehensive data mining with the aid of PPK and ML.

## FUNDING

The study was supported by Bill & Melinda Gates Foundation (INV‐007625), Capital's Funds for Health Improvement and Research (CFH 2022‐2Z‐40917), and Clinical Cohort Construction Program of Peking University Third Hospital (grand number BYSYDL2023004).

## CONFLICT OF INTEREST

All the other authors declared no competing interests.

## AUTHOR CONTRIBUTIONS

W.K., Y.P., Y.W., and H.L. wrote the manuscript. D.L. and W.T. designed the research. W.K., Y.P., Y.W., Y.H., Z.J., X.T., S.B., S.W., F.F., Y.J., J.L., H.Y.L., and Y.W. performed the research; W.K., Y.W., and H.L. analyzed the data.

## Supporting information


Data S1.
