# Supplementary material for: Microdose Cocktail Study Reveals the Activity and Key Influencing Factors of OATP1B, P‐Gp, BCRP, and CYP3A in End‐Stage Renal Disease Patients
Source: Clin Pharmacol Ther. 2025 Jan 10;117(5):1303–12. doi: 10.1002/cpt.3546 (PMC11993298; doi:10.1002/cpt.3546)
Supplement: Supplementary file 1 — Data S1. [file CPT-117-1303-s001.docx]

Microdose cocktail study reveals the activity and key influencing factors of OATP1B, P-gp, BCRP and CYP3A in end-stage renal disease patients

Weijie Kong^1,2#^; Yuejuan Pan^1#^; Yujie Wu^1,2#^; Yiyi Hu^1,2^; Zhenbin Jiang^1^; Xinkui Tian^1^; Shuhong Bi^1^; Song Wang^1^; Feifei Feng^2^; Yuyan Jin^2^; Jiayu Li^1,2^; Haiyan Li^2,3^; Yue Wang^1^; Hao Liang^1,2*^; Wen Tang^1*^; Dongyang Liu^2,3,4*^

^1^Department of Nephrology, Peking University Third Hospital, Beijing, China.

^2^Drug Clinical Trial Center, Peking University Third Hospital, Beijing, China.

^3^Institute of Medical Innovation, Peking University Third Hospital, Beijing, China

^4^Beijing Key Laboratory of Cardiovascular Receptors Research, Peking University Third Hospital, Beijing, China

^#^These authors contributed equally to this work: Weijie Kong, Yuejuan Pan, Yujie Wu

^*^Correspondence:

Dongyang Liu

liudongyang@vip.sina.com

Wen Tang

tanggwen@126.com

Hao Liang

lianghao86@126.com

**Supplementary Methods**

**Microdose cocktail regimen preparation**

MDZ solution (Jiangsu Nhwa Pharmaceutical Co., LTD, 5 mg/mL), DABE capsule (Boehringer Ingelheim International GmbH, 110 mg), PTV tablet (China Resources Double-Crane Pharmaceutical Co., Ltd, 2 mg), RSV tablet (Astra Zeneca Pharmaceutical Co., Ltd, 10 mg) and ATV tablet (Pfizer Inc. 20 mg) were provided by Peking University Third Hospital. Each tablet or capsule content was ground into powder separately. The powders of DABE, PTV, RSV and ATV were dissolved into sterile saline solution in volumes of 110, 100, 100 and 100 mL, respectively. For MDZ, 0.5 mL of the solution was extracted and then diluted with saline to concentration of 0.05 mg/mL. Finally, the parent solutions of MDZ, DABE, PTV, RSV and ATV (0.2, 0.375, 0.5, 0.5 and 0.5 mL, respectively) were taken and diluted with 50 mL of saline.

**Bioanalysis**

After blood sample collection, 1 M ammonium acetate (pH 5.0) was added to each statin sample at a ratio of 5:100 in advance to prevent the interconversion between statins and metabolites. The samples were stored at -80℃ before analysis. Plasma concentrations of substrate drugs were quantified using validated liquid chromatography coupled with mass spectrometer (LC-MS, Shimadzu) according to previous established method^[1]^. The assay linearity was evaluated between 0.5-256 pg/mL for MDZ, 10-5120 pg/mL for DAB, and 1-1000 pg/mL for statins, respectively. The intra-day and inter-day precision and accuracy were assessed in blank human EDTA plasma at LLOQ, low, medium, and high QC concentrations. Stability was assessed using QC samples under on-bench, on-machine, and freeze-thaw conditions.

**Plasma protein binding determination**

Plasma protein binding was assessed using ultrafiltration with Vivacon 500 Hydrosart device (30,000 MWCO, Sartorius Stedim Lab Ltd., Sperry Way, Stonehouse, UK). EDTA plasma samples (n = 3), spiked with the test compounds, were prepared, and 400 µL of each sample was loaded into the ultrafiltration units. The samples were then centrifuged at 12,000 g for 40 minutes at 4°C. Following centrifugation, aliquots of the collected filtrates and corresponding initial plasma samples were diluted. The diluted samples were then analyzed by validated LC-MS/MS method.

1. Chavez-Eng CM, Lutz RW, Goykhman D, Bateman KP. Microdosing Cocktail Assay Development for Drug-Drug Interaction Studies. J Pharm Sci. 2018. 107(7): 1973-1986.

**
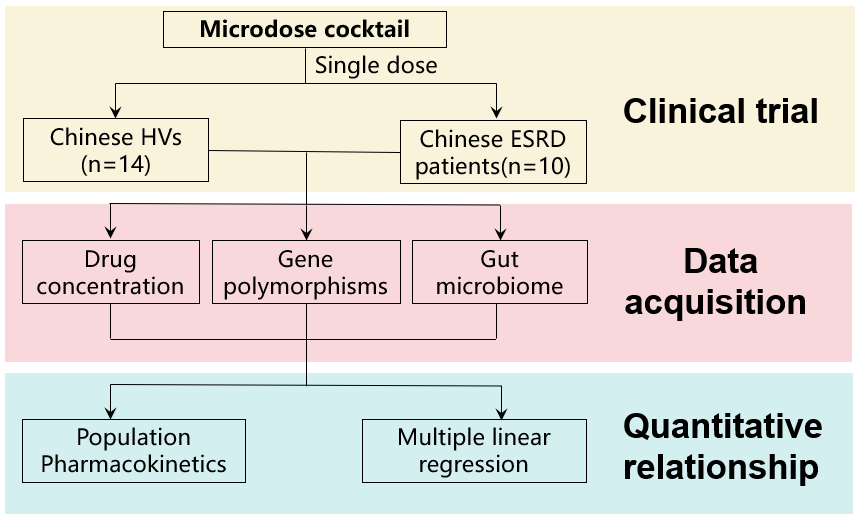
**

**Supplementary Figure S1.** Workflow of this study.


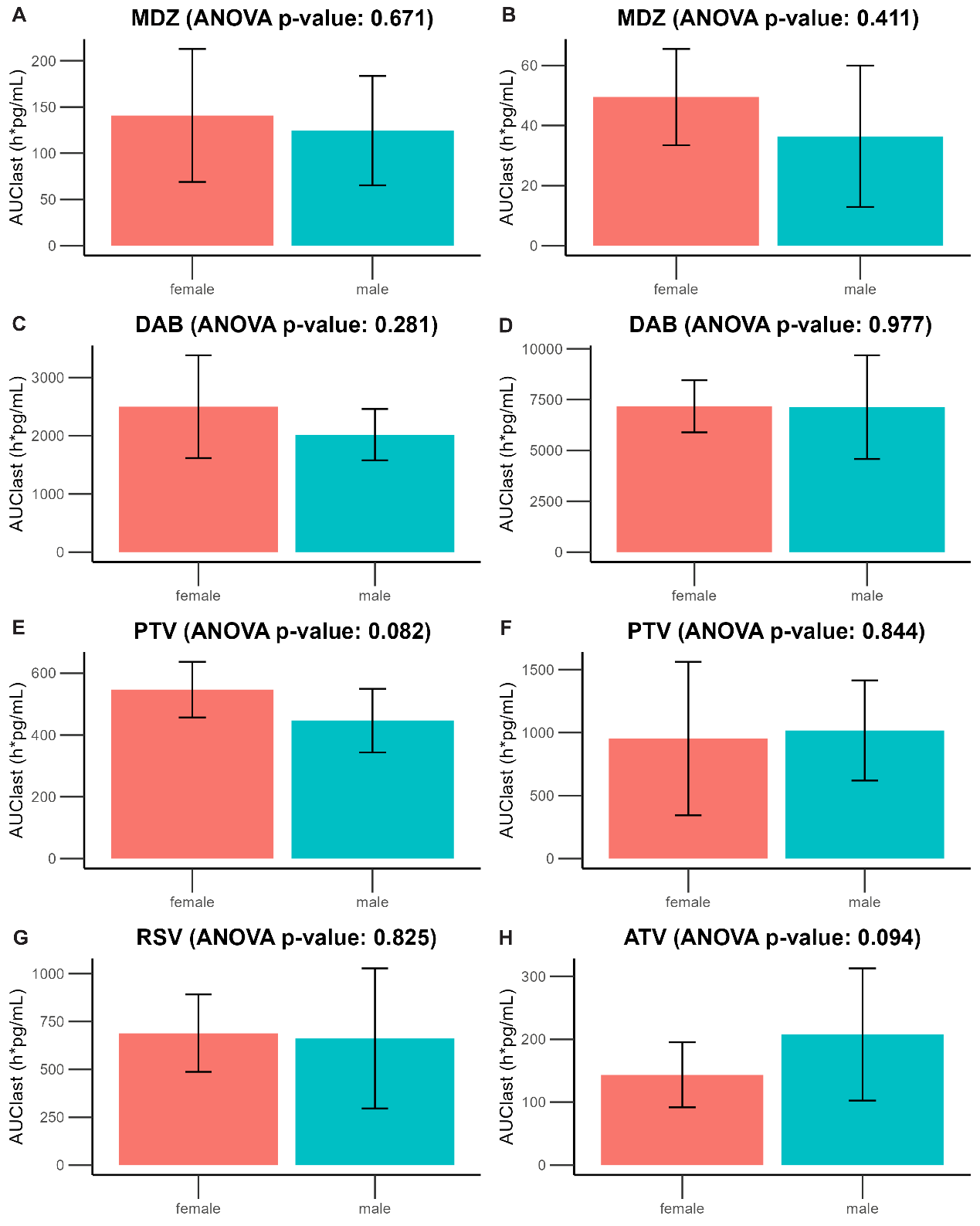


**Supplementary Figure S2.** Female (red) and male (blue) exposure of MDZ (A), DAB (C), PTV (E) in HVs and MDZ (B), DAB (D), PTV (F) in ESRD patients, respectively. Female (red) and male (blue) exposure of RSV (G) and ATV (H) was shown for all subjects.


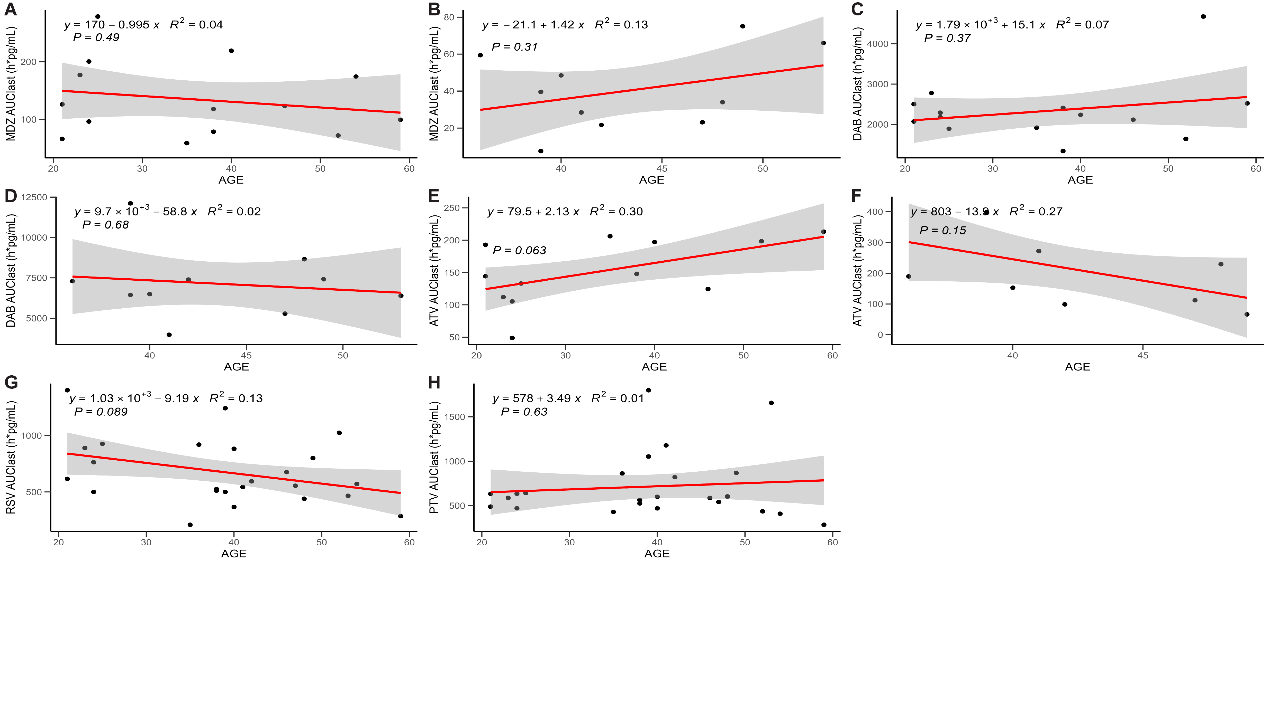


**Supplementary Figure S3.** Correlations of age and drug exposure of MDZ (A), DAB (C), PTV (E) in ESRD HVs and MDZ (B), DAB (D), PTV (F) in ESRD patients, and RSV (G) and ATV (H) for all subjects, respectively.


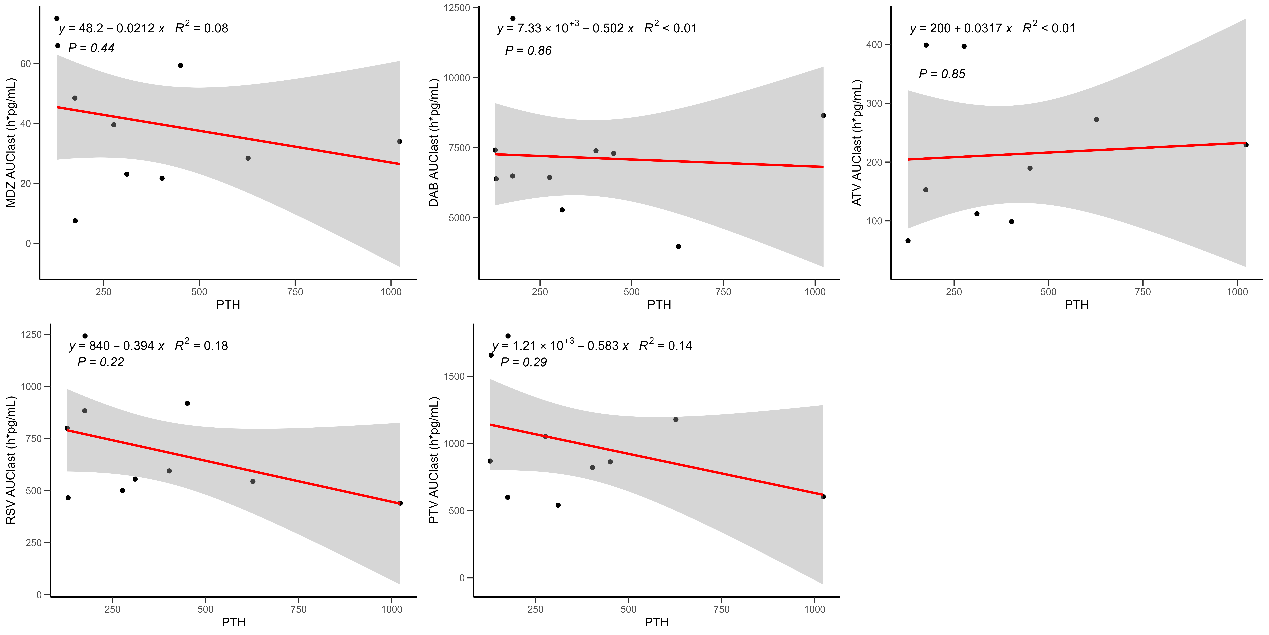


**Supplementary Figure S4.** Correlations of PTH level and drug exposure.


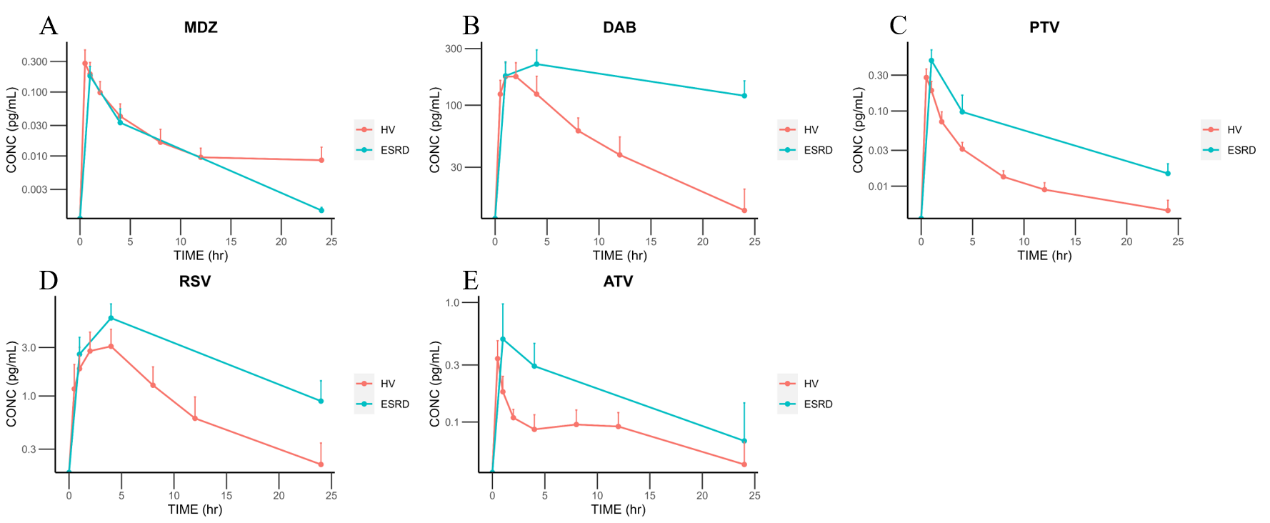


**Supplementary Figure S5.** Concentration-time curve of MDZ (A), DAB (B), PTV (C), RSV (D) and ATV (E). The data of HV and ESRD are displayed as red and blue line, respectively.


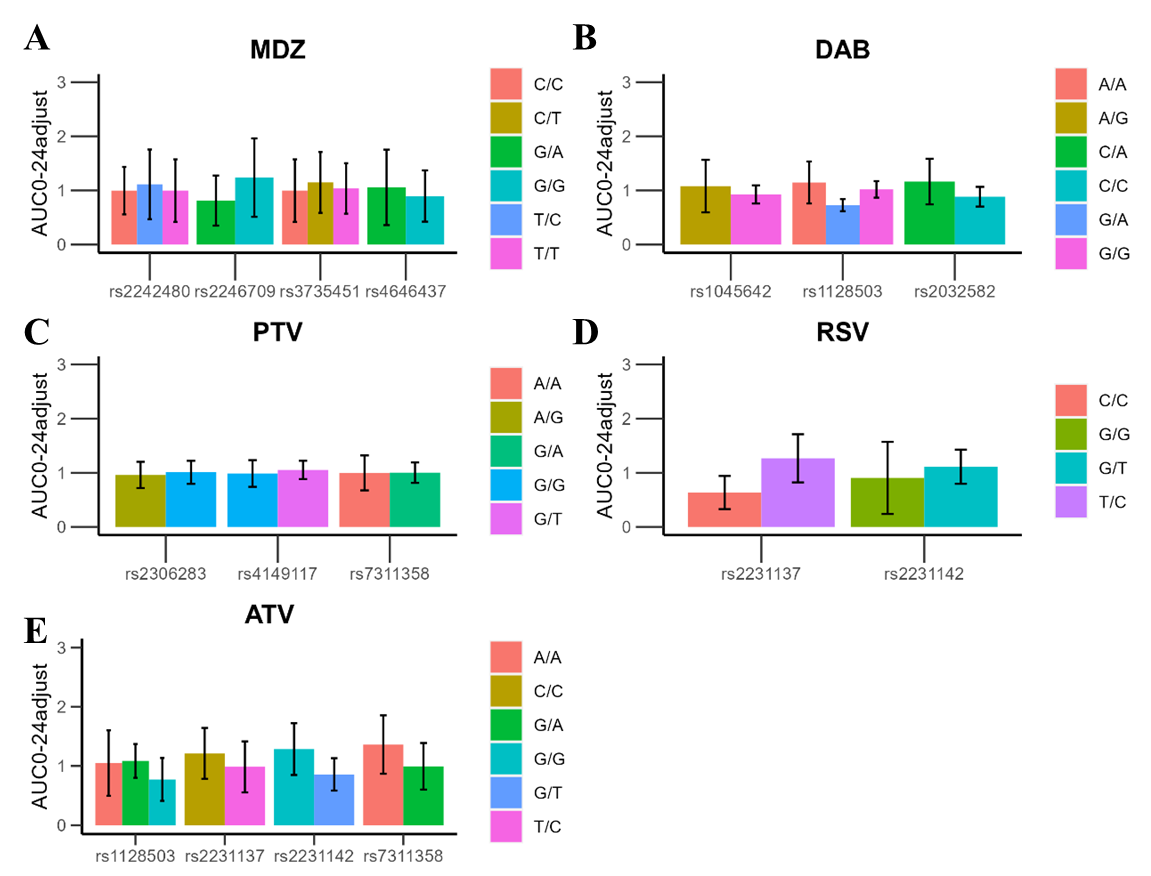


**Supplementary Figure S6.** Exposure of MDZ (A), DAB (B), PTV (C), RSV (D) and ATV (E) in HVs with specific SNP.


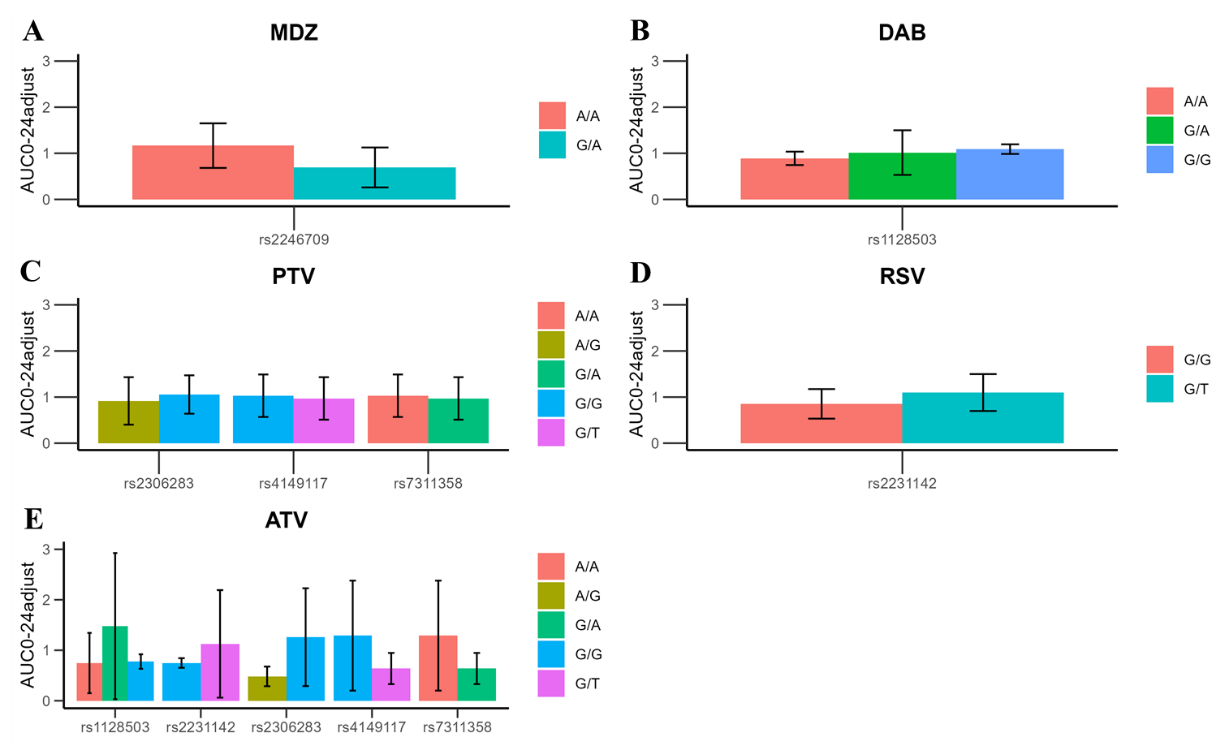


**Supplementary Figure S7.** Exposure of MDZ (A), DAB (B), PTV (C), RSV (D) and ATV (E) in ESRD patients with specific SNP.


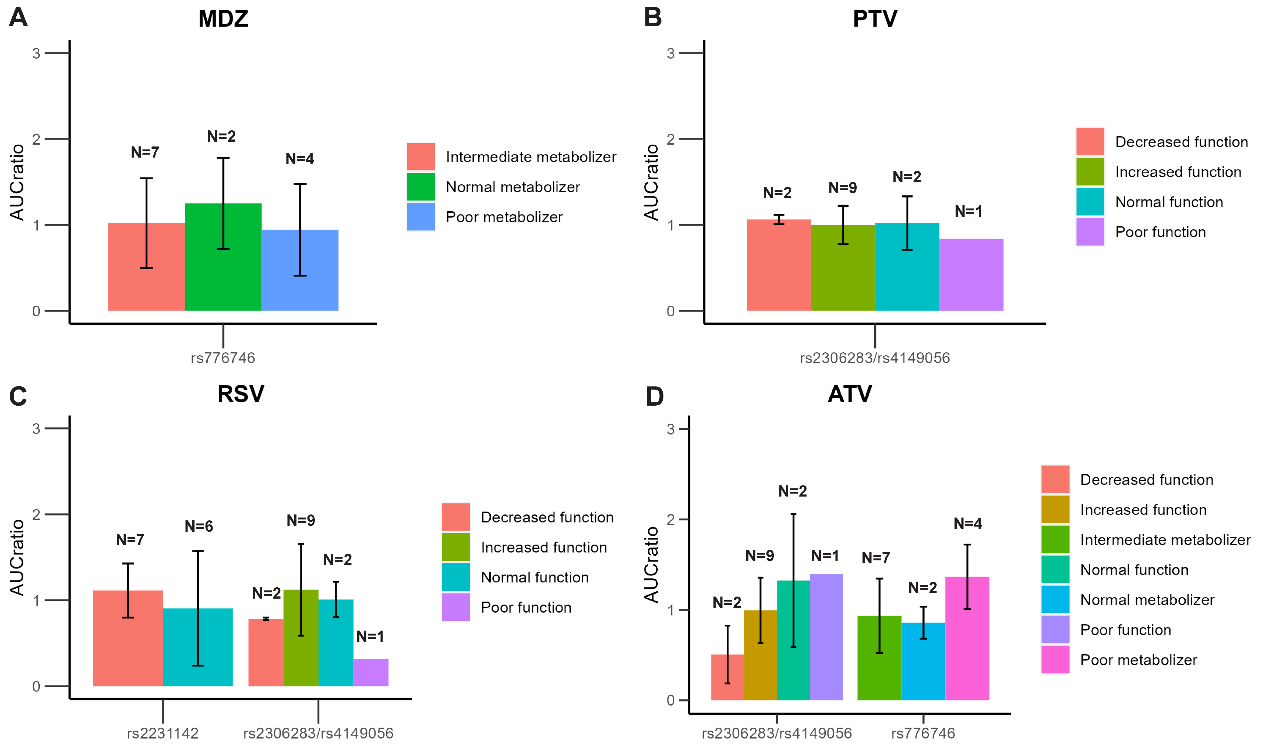


**Supplementary Figure S8.** Exposure of MDZ (A), PTV (B), RSV (C) and ATV (D) in HVs with specific phenotype.


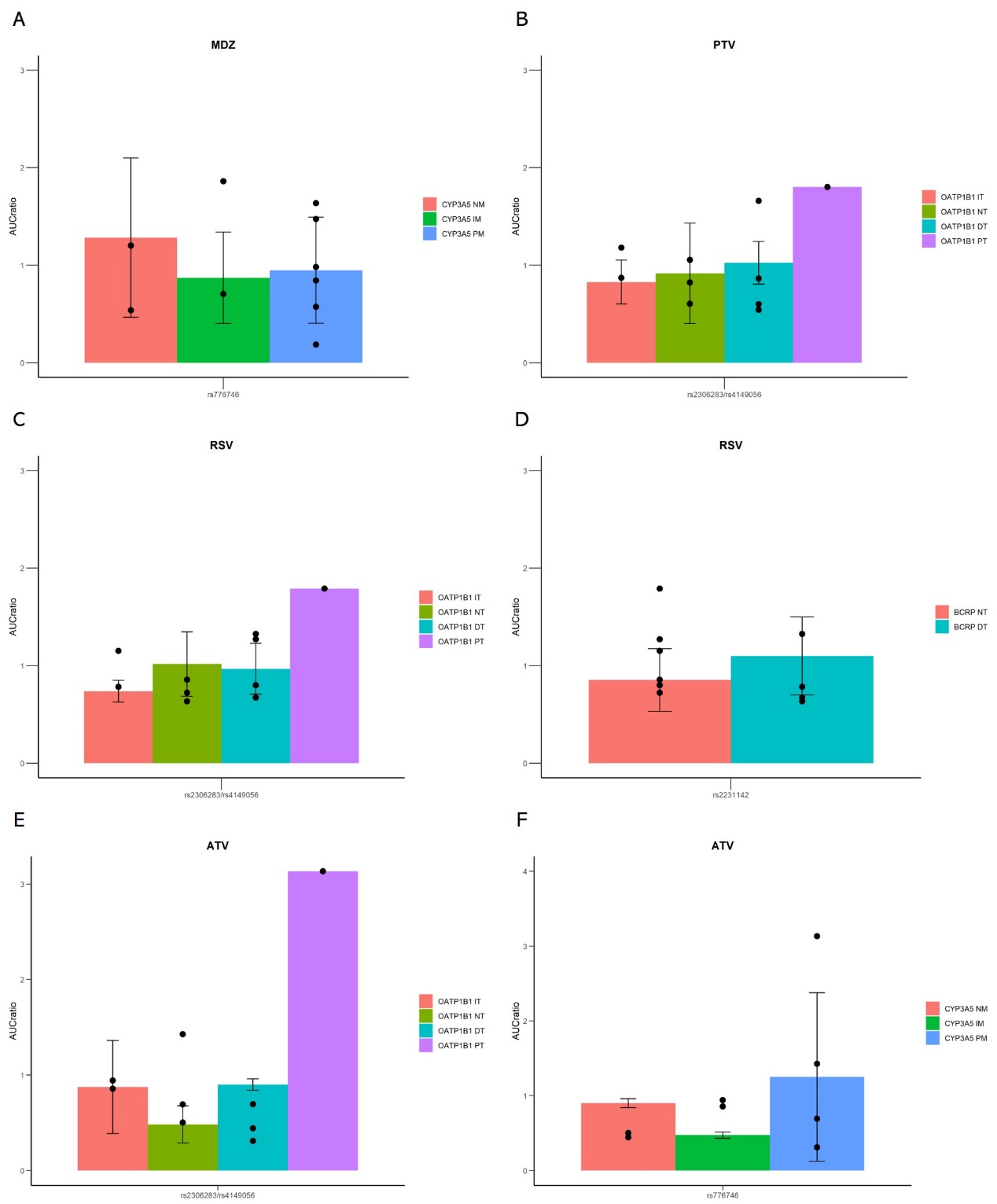


**Supplementary Figure S9.** Exposure of MDZ, PTV, RSV and ATV in ESRD patients with specific phenotype. The individual data is shown as black dots. IT: increased function; NT: normal function; DT: decreased function; PT: poor function; NM: normal metabolizers; IM: intermediate metabolizers; PM: poor metabolizers.

**
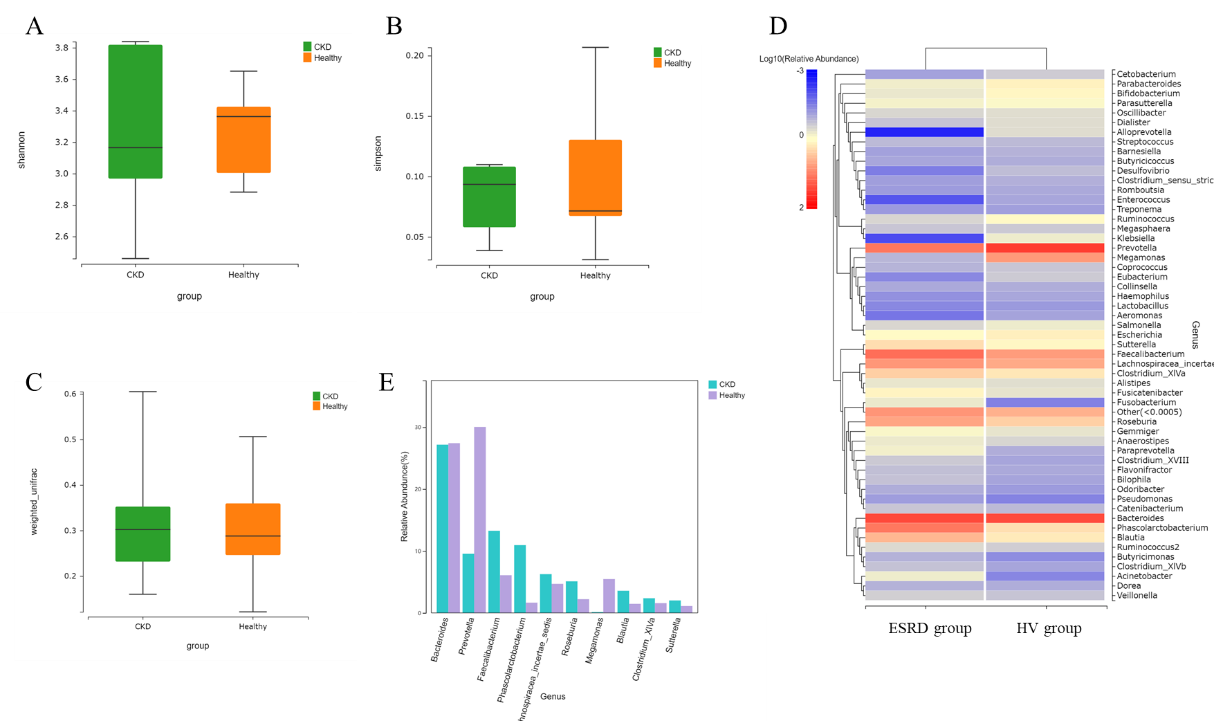
**

**Supplementary Figure S10.** Gut microbiome analysis of HVs and ESRD patients. The α-diversity was calculated by Shannon (A) and Simpson index (B), while β-diversity was evaluated by weighted unifraction (C). The differential genus between HVs and ESRD patients are displayed as heatmap (D), while the most abundant genus was determined through comparison of the histograms (E).


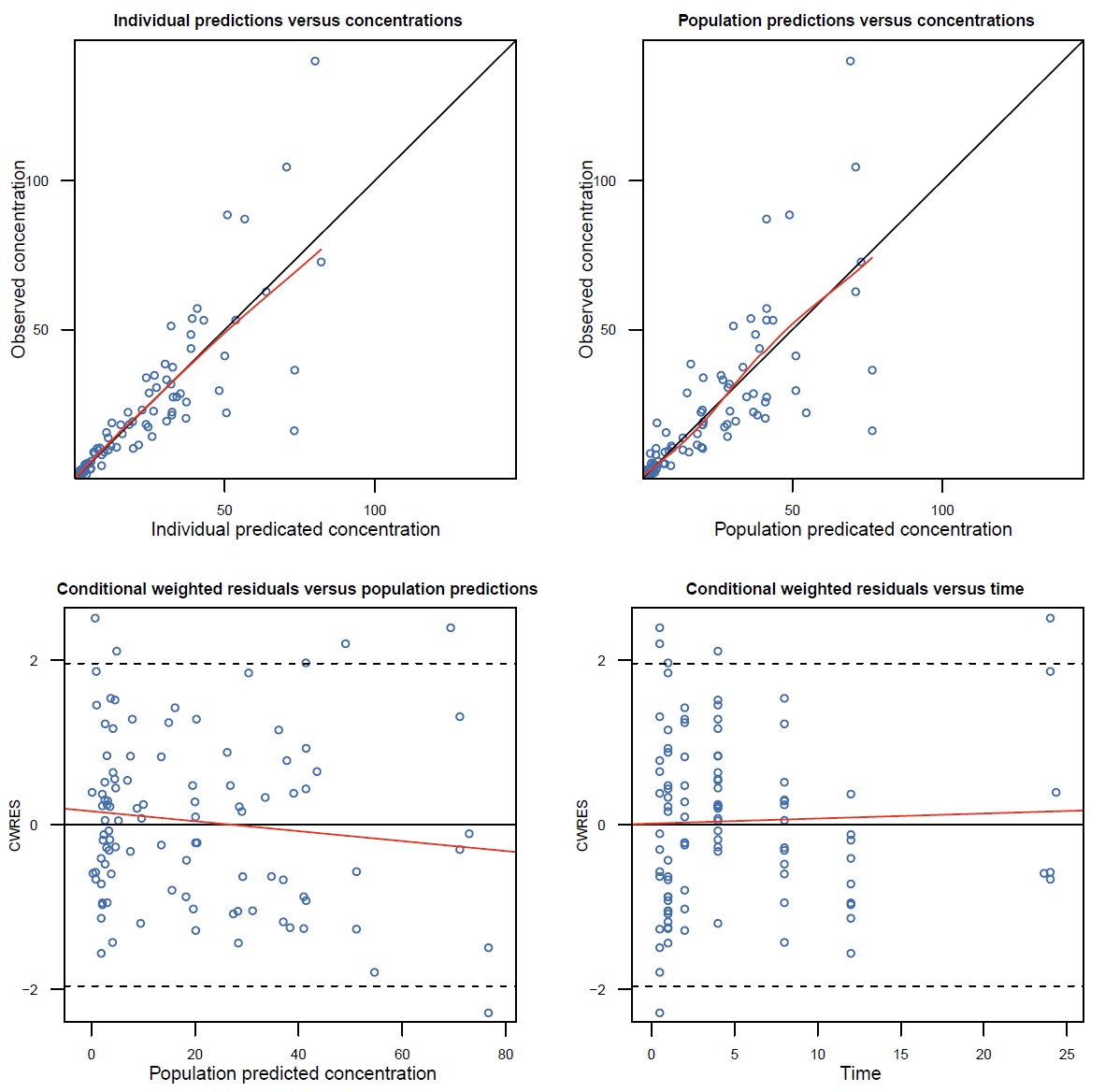


**Supplementary Figure S11.** Goodness-of-fit plots for the final PPK model of MDZ. The red dotted line represents the fit of a linear regression through the data, with the blue circles representing the observed data.


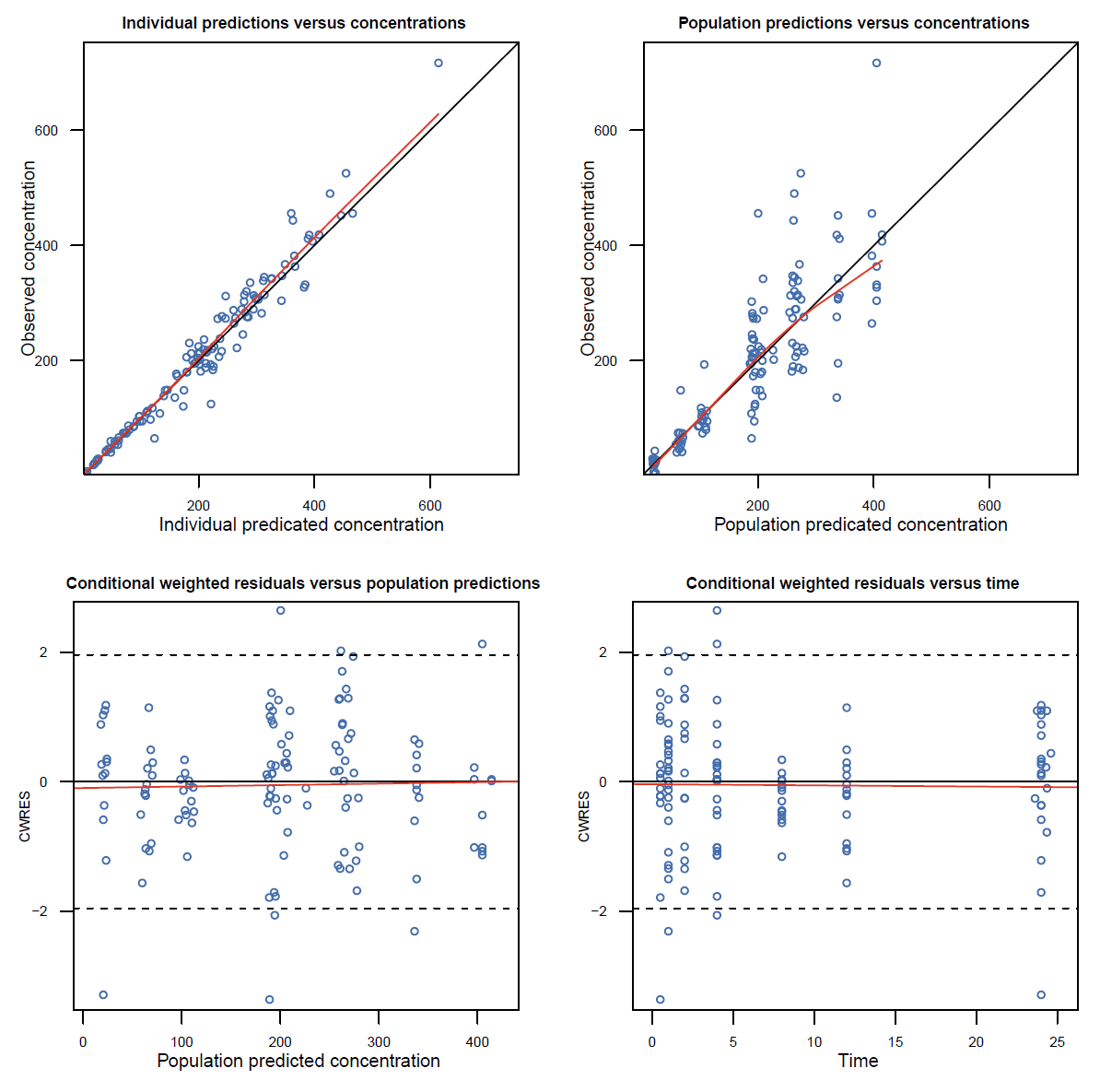


**Supplementary Figure S12.** Goodness-of-fit plots for the final PPK model of DAB. The red dotted line represents the fit of a linear regression through the data, with the blue circles representing the observed data.


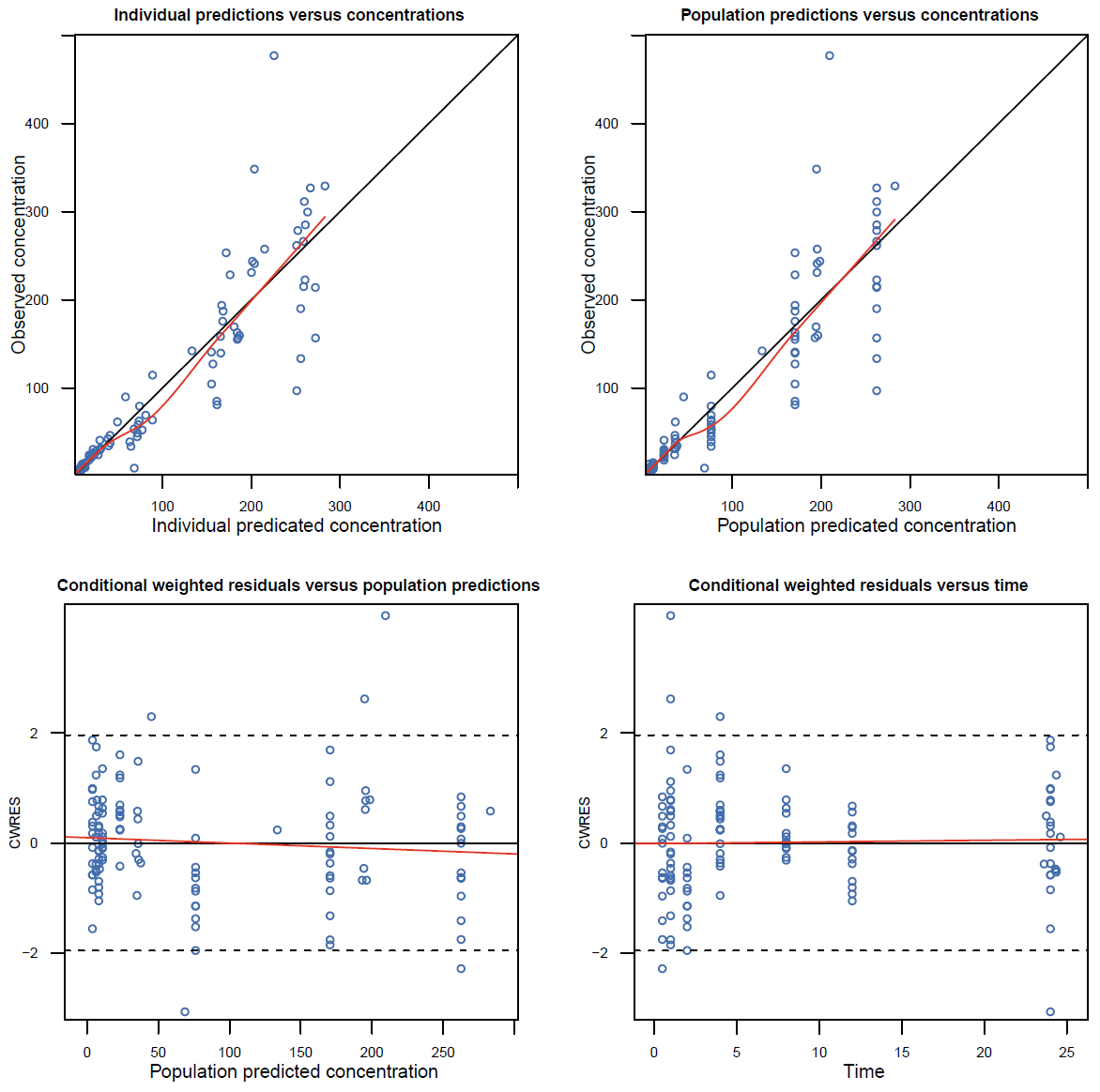


**Supplementary Figure S13.** Goodness-of-fit plots for the final PPK model of PTV. The red dotted line represents the fit of a linear regression through the data, with the blue circles representing the observed data.


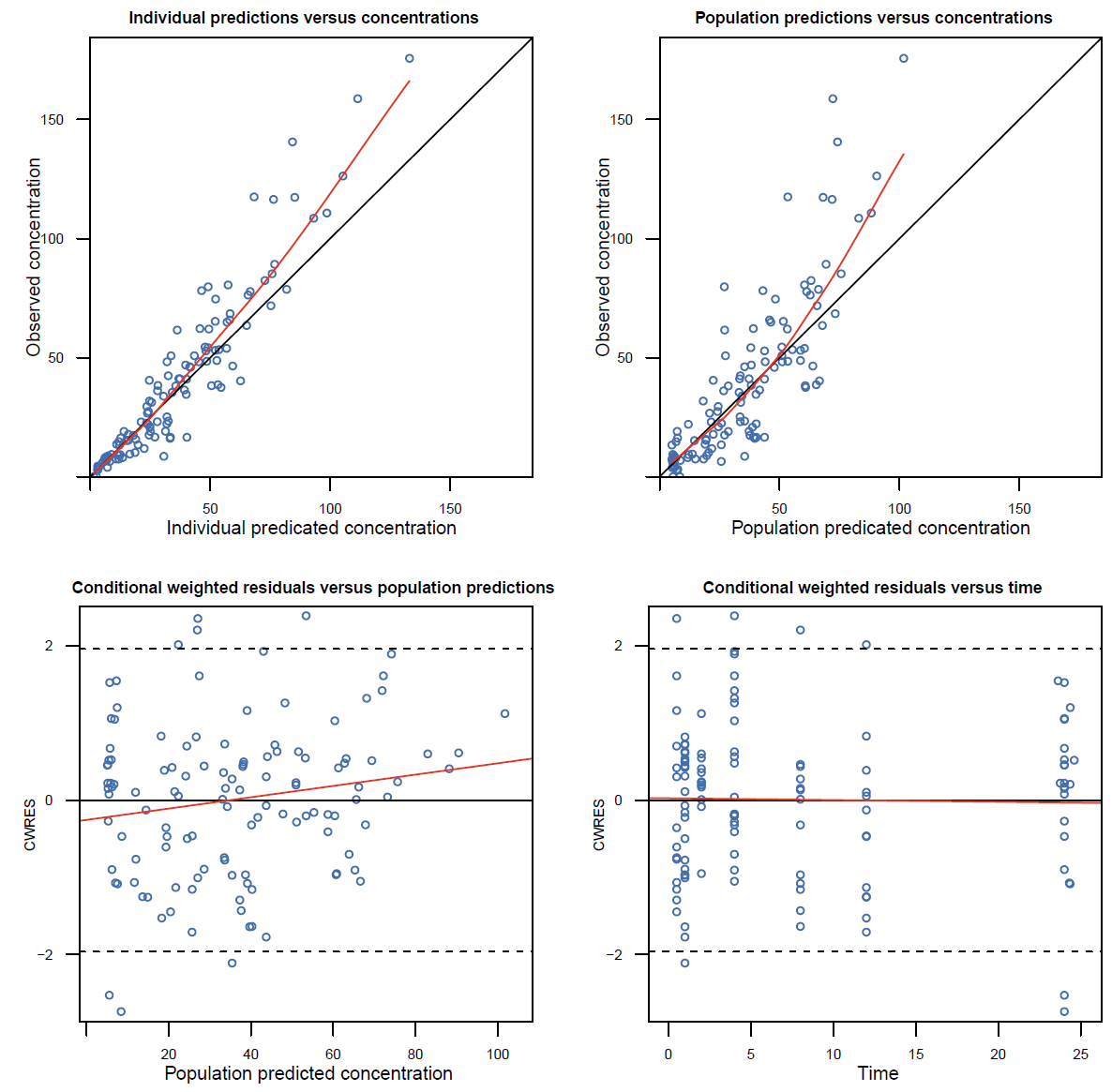


**Supplementary Figure S14.** Goodness-of-fit plots for the final PPK model of RSV. The red dotted line represents the fit of a linear regression through the data, with the blue circles representing the observed data.


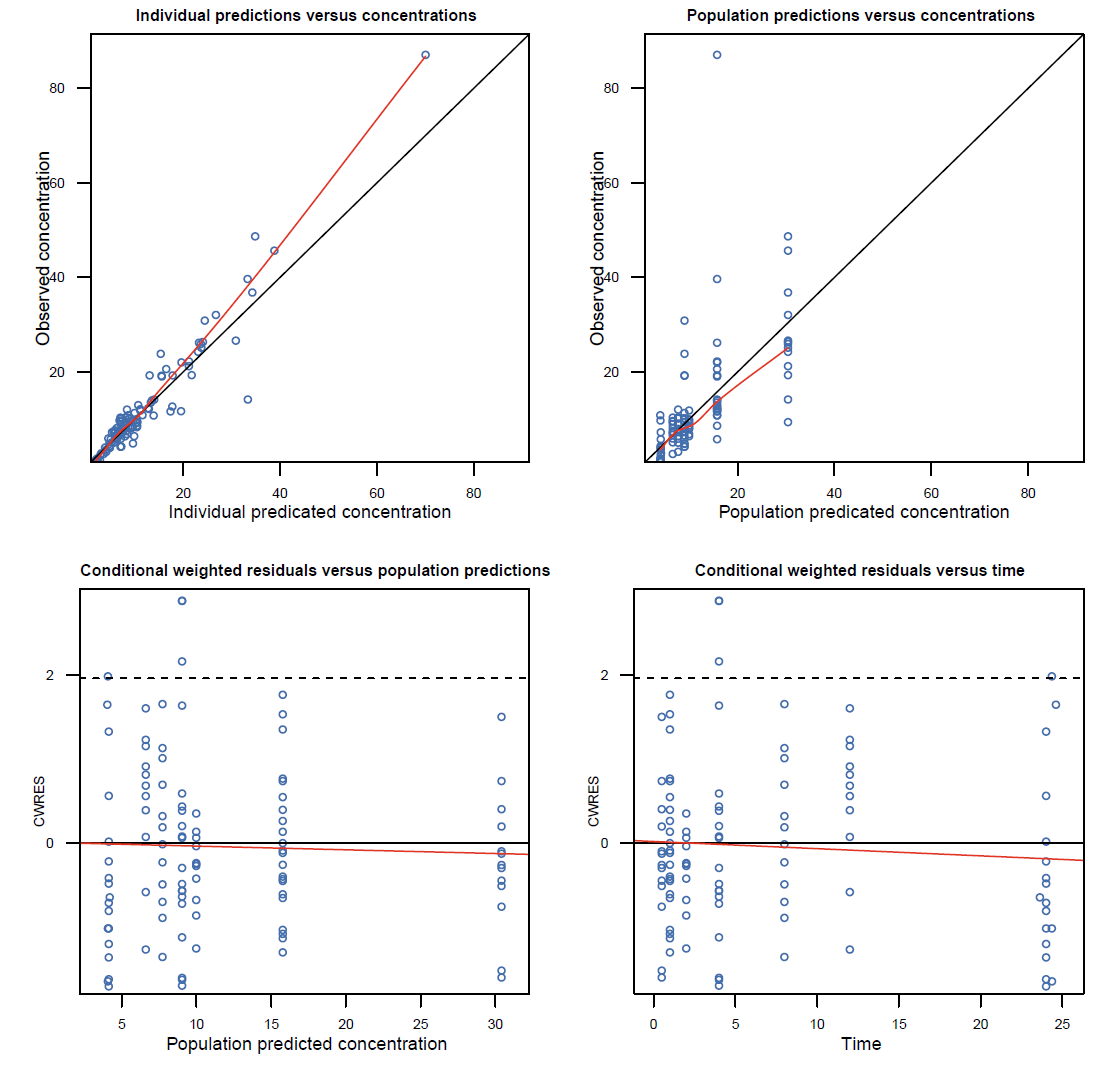


**Supplementary Figure S15.** Goodness-of-fit plots for the final PPK model of ATV. The red dotted line represents the fit of a linear regression through the data, with the blue circles representing the observed data.


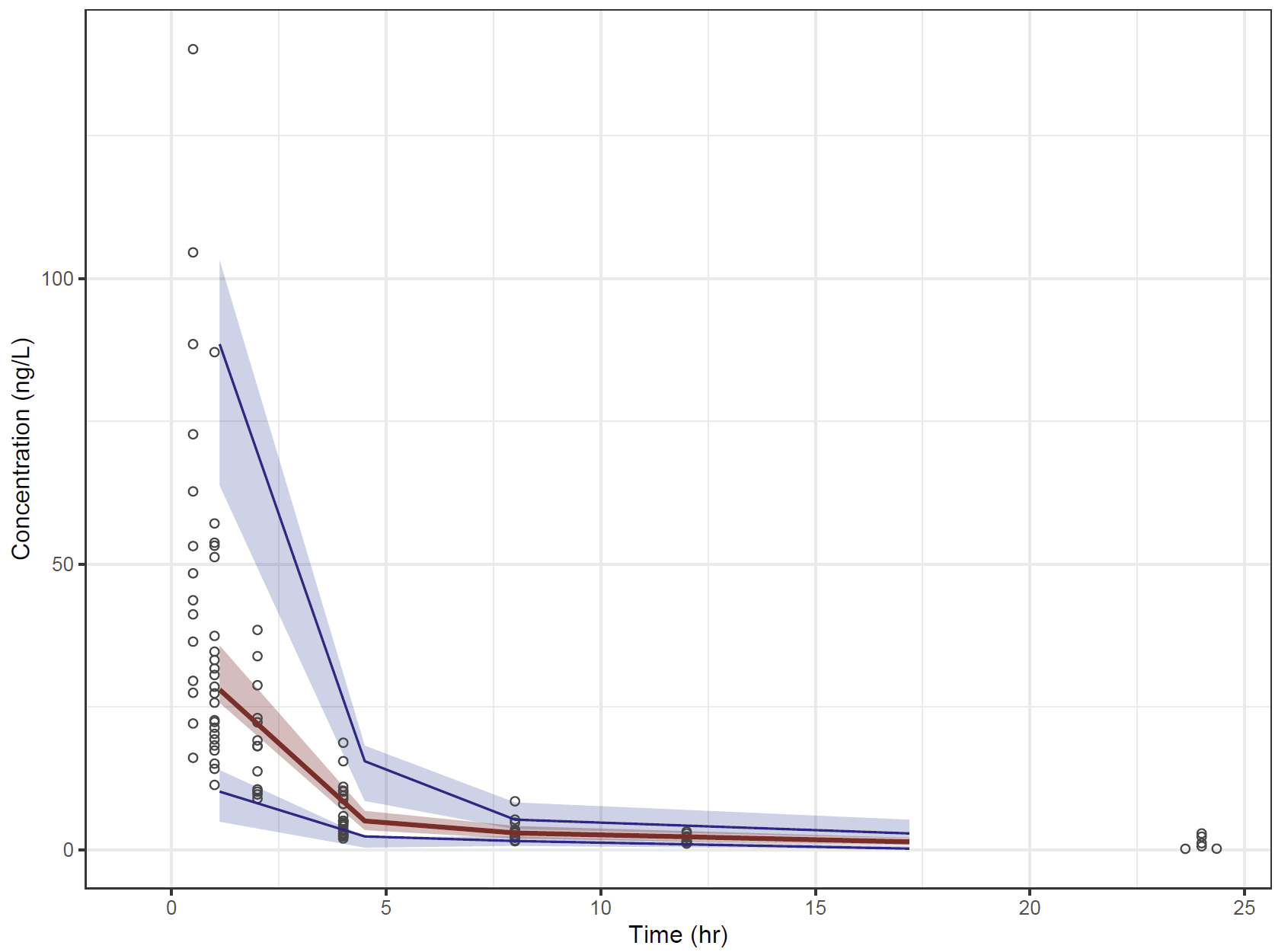


**Supplementary Figure S16.** Prediction-corrected visual predictive checks for the final population PK model for all data with MDZ. The lower and upper blue lines represent the 5th and 95th percentiles for the observed data. The red line represents the 50th percentile for the observed data. The shaded areas represent the 90% confidence intervals for the 5th, 50th, and 95th percentiles of the simulated data.


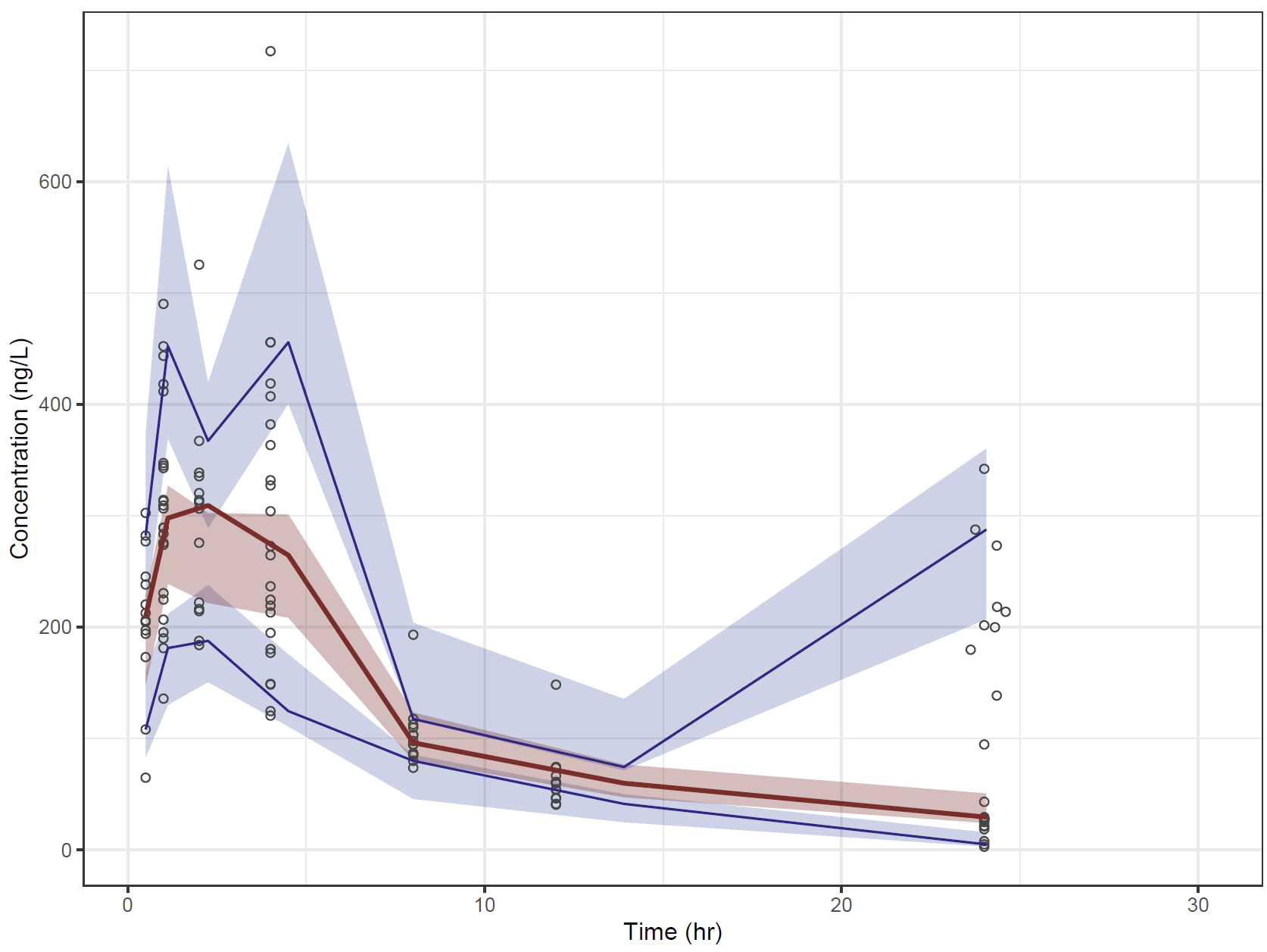


**Supplementary Figure S17.** Prediction-corrected visual predictive checks for the final population PK model for all data with DAB. The lower and upper blue lines represent the 5th and 95th percentiles for the observed data. The red line represents the 50th percentile for the observed data. The shaded areas represent the 90% confidence intervals for the 5th, 50th, and 95th percentiles of the simulated data.


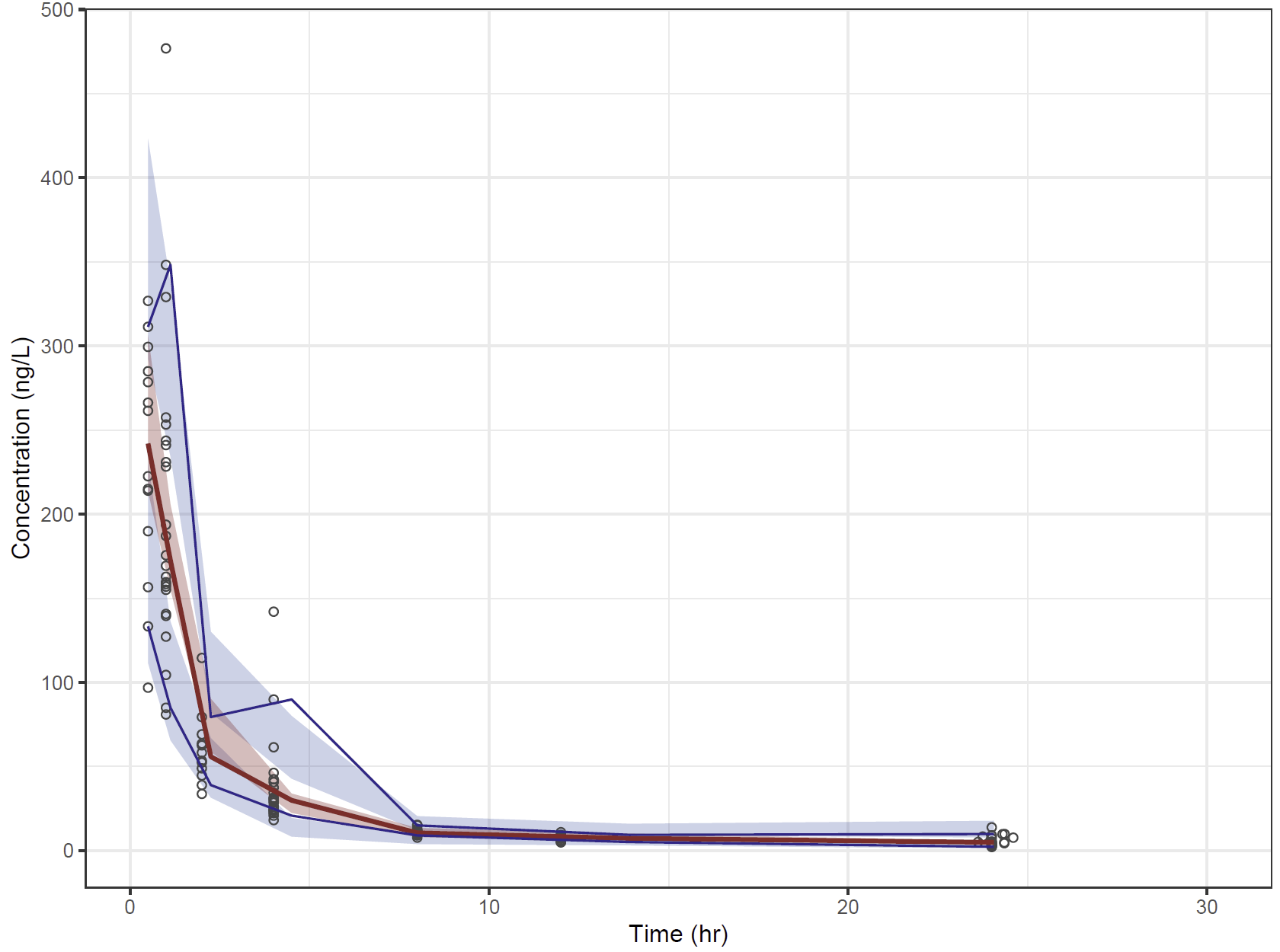


**Supplementary Figure S18.** Prediction-corrected visual predictive checks for the final population PK model for all data with PTV. The lower and upper blue lines represent the 5th and 95th percentiles for the observed data. The red line represents the 50th percentile for the observed data. The shaded areas represent the 90% confidence intervals for the 5th, 50th, and 95th percentiles of the simulated data.


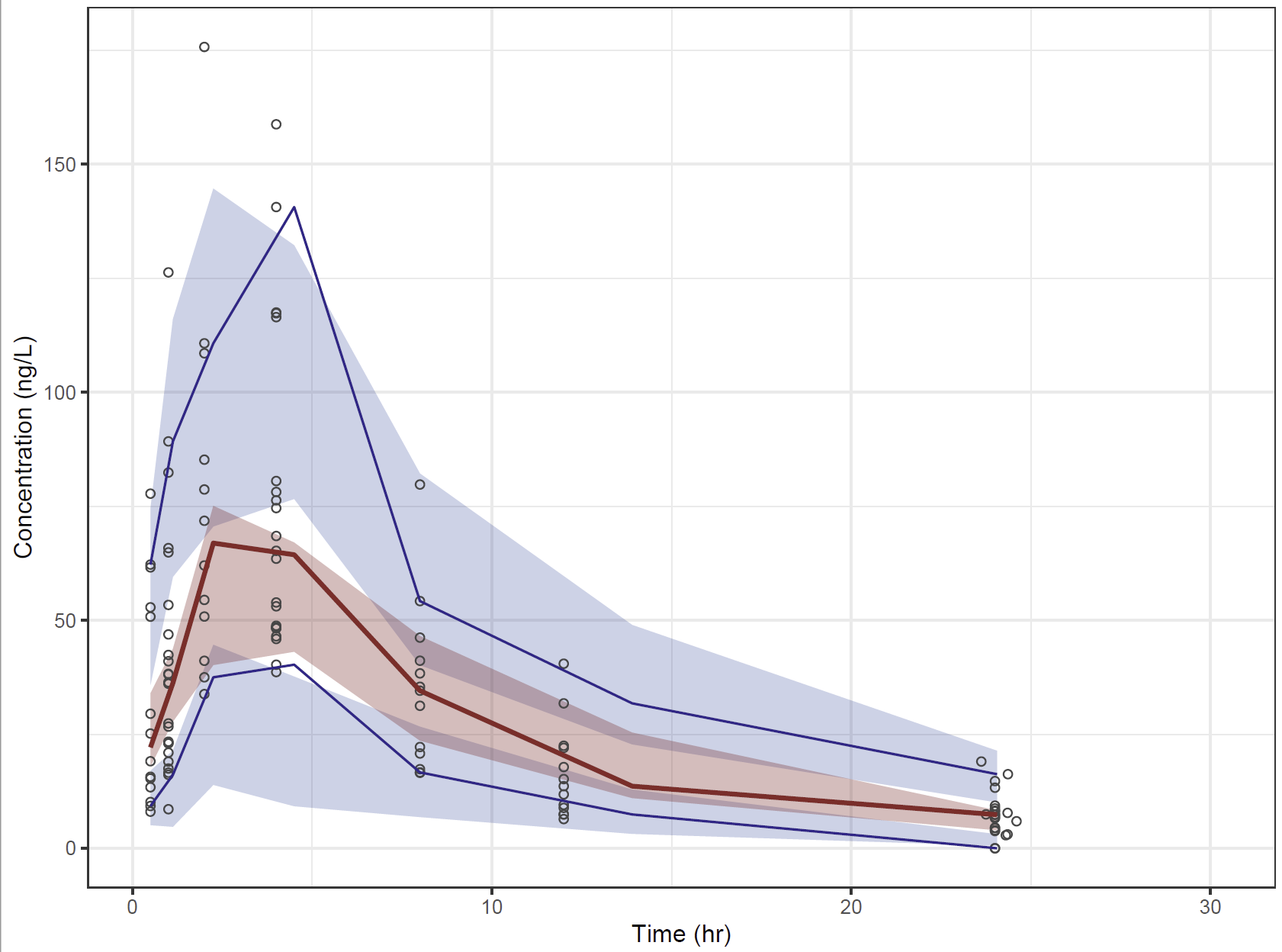


**Supplementary Figure S19.** Prediction-corrected visual predictive checks for the final population PK model for all data with RSV. The lower and upper blue lines represent the 5th and 95th percentiles for the observed data. The red line represents the 50th percentile for the observed data. The shaded areas represent the 90% confidence intervals for the 5th, 50th, and 95th percentiles of the simulated data.


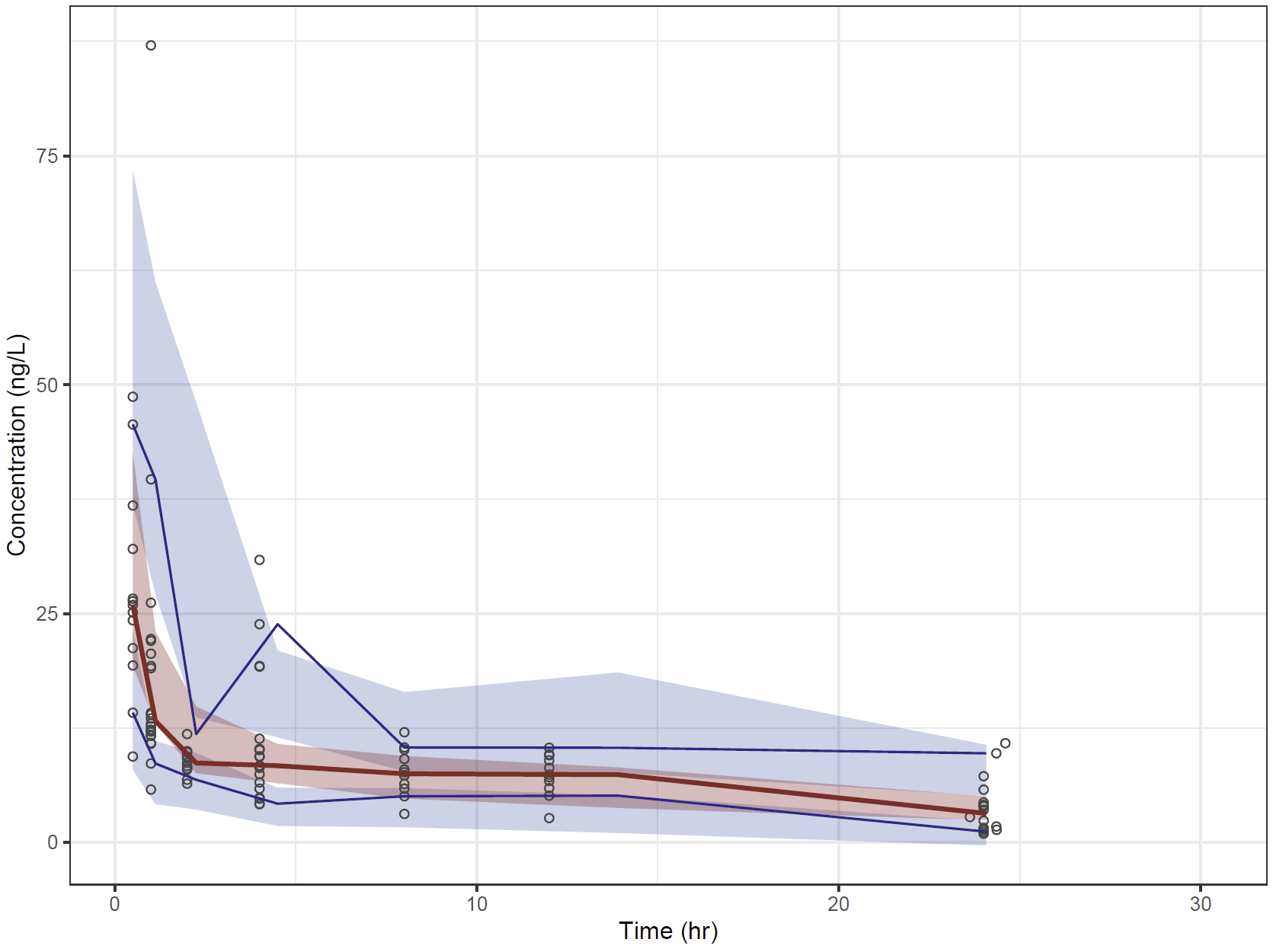


**Supplementary Figure S20.** Prediction-corrected visual predictive checks for the final population PK model for all data with ATV. The lower and upper blue lines represent the 5th and 95th percentiles for the observed data. The red line represents the 50th percentile for the observed data. The shaded areas represent the 90% confidence intervals for the 5th, 50th, and 95th percentiles of the simulated data.


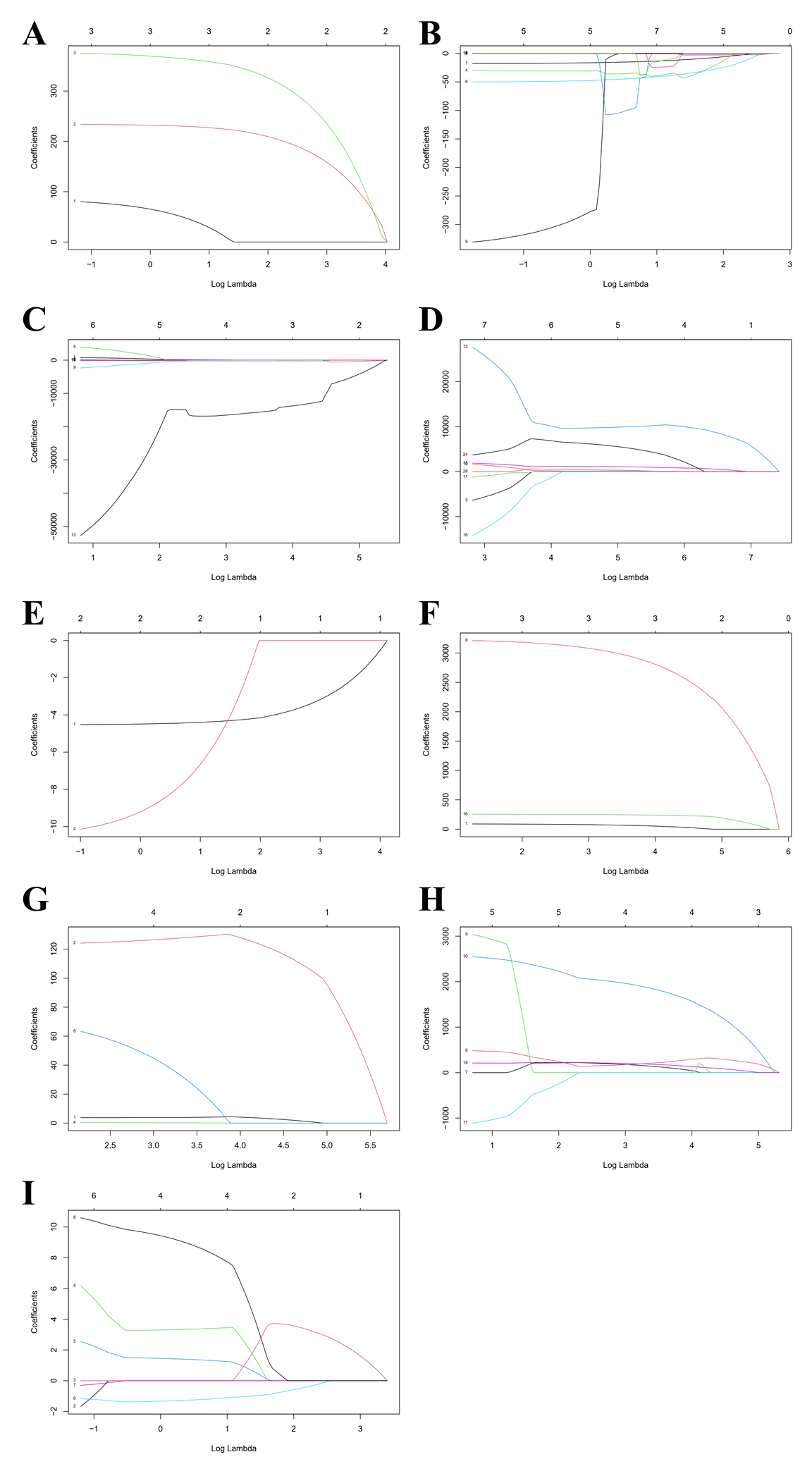


**Supplementary Figure S21.** LASSO coefficient profiles of the variables for MDZ (A), DAB (C), PTV (E), RSV (G) and ATV (I) in HVs, and MDZ (B), DAB (D), PTV (F) and RSV (H) in ESRD population.


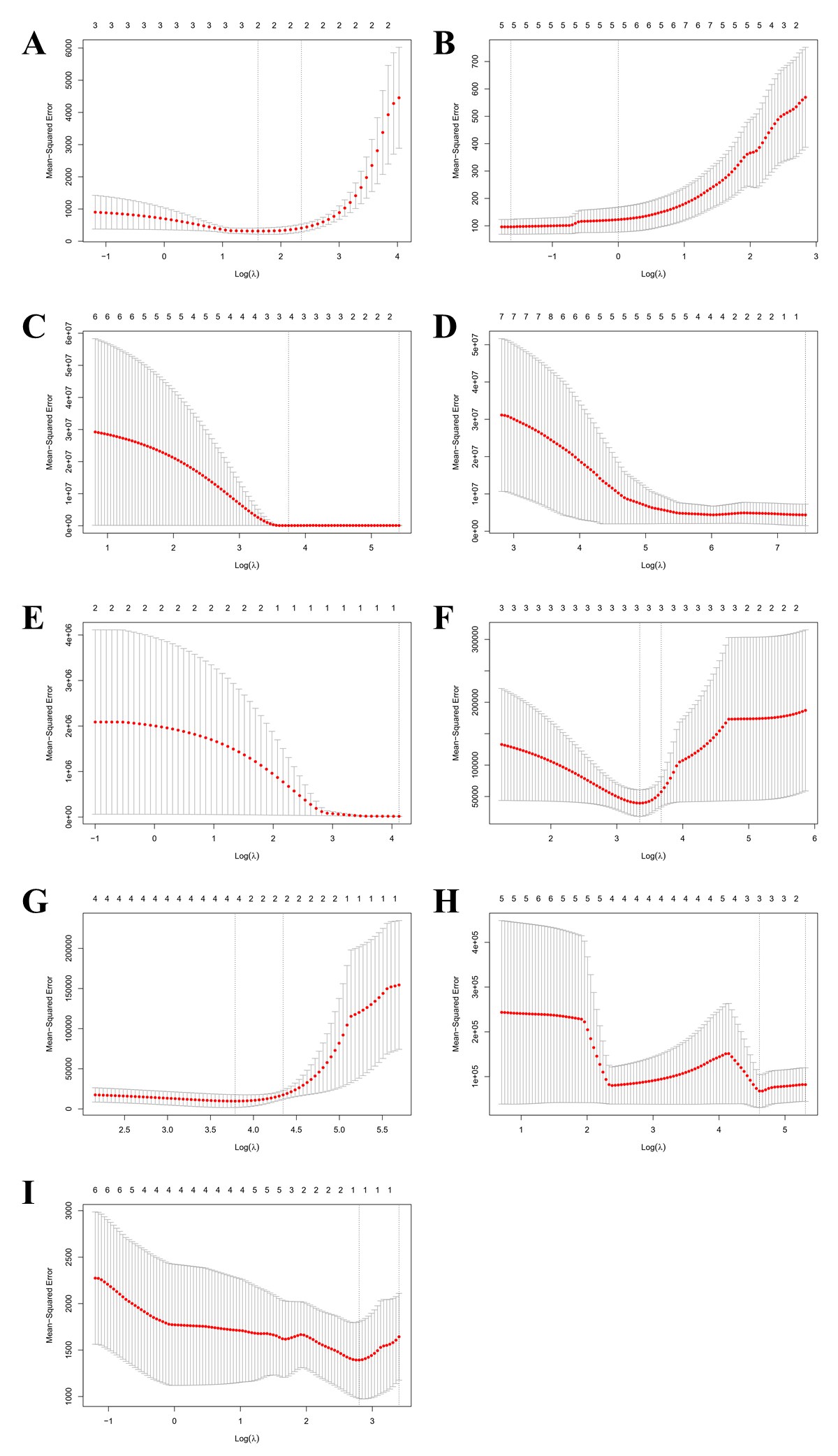


**Supplementary Figure S22.** Tuning parameter (λ) selection in the LASSO model used 10-fold cross-validation via minimum criteria for MDZ (A), DAB (C), PTV (E), RSV (G) and ATV (I) in HVs, and MDZ (B), DAB (D), PTV (F) and RSV (H) in ESRD population.

**Supplementary Table S1** Precision and accuracy test of all five drugs

| **MDZ** | | | | | |
| --- | --- | --- | --- | --- | --- |
| Variable | | LLOQ | Low QC | Medium QC | High QC |
| Nominal Concentration (pg/mL) | | 0.5 | 1.5 | 20.0 | 200.0 |
| Intra-day test batch 1  (n = 6) | Mean | 0.5 | 1.5 | 19.6 | 186.4 |
|  | Precision (CV) (%) | 11.7 | 13.4 | 5.6 | 2.7 |
|  | Accuracy (%) | 4.2 | -0.6 | -2.2 | -6.8 |
| Intra-day test batch 2  (n = 6) | Mean | 0.6 | 1.4 | 20.3 | 212.5 |
|  | Precision (CV) (%) | 11.5 | 8.0 | 10.4 | 11.0 |
|  | Accuracy (%) | 16.9 | -9.1 | 1.5 | 6.2 |
| Intra-day test batch 3  (n = 6) | Mean | 0.5 | 1.5 | 21.1 | 188.5 |
|  | Precision (CV) (%) | 8.5 | 7.1 | 9.4 | 4.2 |
|  | Accuracy (%) | 0.3 | -1.9 | 5.6 | -5.7 |
| Inter-day test  (n = 18) | Mean | 0.5 | 1.4 | 20.3 | 195.8 |
|  | Precision (CV) (%) | 12.3 | 9.9 | 8.9 | 9.3 |
|  | Accuracy (%) | 7.5 | -4.1 | 1.6 | -2.1 |
| **DAB** | | | | | |
| Variable | | LLOQ | Low QC | Medium QC | High QC |
| Nominal Concentration (pg/mL) | | 10.0 | 30.0 | 400.0 | 4000.0 |
| Intra-day test batch 1  (n = 6) | Mean | 10.8 | 27.8 | 398.0 | 4262.0 |
|  | Precision (CV) (%) | 15.0 | 5.3 | 3.0 | 2.7 |
|  | Accuracy (%) | 8.0 | -7.3 | -0.5 | 6.6 |
| Intra-day test batch 2  (n = 6) | Mean | 9.9 | 30.2 | 390.6 | 4021.9 |
|  | Precision (CV) (%) | 14.0 | 9.1 | 4.5 | 3.0 |
|  | Accuracy (%) | -0.7 | 0.7 | -2.3 | 0.5 |
| Intra-day test batch 3  (n = 6) | Mean | 10.3 | 31.5 | 394.9 | 4019.3 |
|  | Precision (CV) (%) | 7.9 | 5.1 | 3.8 | 2.9 |
|  | Accuracy (%) | 3.1 | 5.0 | -1.3 | 0.5 |
| Inter-day test  (n = 18) | Mean | 10.3 | 29.8 | 394.5 | 4101.1 |
|  | Precision (CV) (%) | 12.5 | 8.3 | 3.6 | 3.9 |
|  | Accuracy (%) | 3.5 | -0.5 | -1.4 | 2.5 |
| **PTV** | | | | | |
| Variable | | LLOQ | Low QC | Medium QC | High QC |
| Nominal Concentration (pg/mL) | | 1.0 | 3.0 | 48.0 | 800.0 |
| Intra-day test batch 1  (n = 6) | Mean | 1.1 | 2.7 | 44.0 | 702.2 |
|  | Precision (CV) (%) | 17.6 | 9.5 | 4.1 | 8.7 |
|  | Accuracy (%) | 6.7 | -10.1 | -8.3 | -12.2 |
| Intra-day test batch 2  (n = 6) | Mean | 1.1 | 3.0 | 47.5 | 821.4 |
|  | Precision (CV) (%) | 9.7 | 9.8 | 2.7 | 2.0 |
|  | Accuracy (%) | 11.3 | -0.7 | -1.1 | 2.7 |
| Intra-day test batch 3  (n = 6) | Mean | 0.9 | 2.9 | 52.0 | 864.3 |
|  | Precision (CV) (%) | 11.3 | 5.7 | 0.9 | 1.8 |
|  | Accuracy (%) | -7.7 | -2.7 | 8.4 | 8.0 |
| Inter-day test  (n = 18) | Mean | 1.0 | 2.9 | 47.9 | 795.9 |
|  | Precision (CV) (%) | 15.0 | 9.1 | 7.7 | 9.9 |
|  | Accuracy (%) | 3.4 | -4.5 | -0.3 | -0.5 |
| **RSV** | | | | | |
| Variable | | LLOQ | Low QC | Medium QC | High QC |
| Nominal Concentration (pg/mL) | | 1.0 | 3.0 | 48.0 | 800.0 |
| Intra-day test batch 1  (n = 6) | Mean | 1.1 | 2.9 | 46.7 | 772.4 |
|  | Precision (CV) (%) | 19.4 | 8.2 | 7.4 | 2.3 |
|  | Accuracy (%) | 9.3 | -2.7 | -2.7 | -3.4 |
| Intra-day test batch 2  (n = 6) | Mean | 0.9 | 2.8 | 48.5 | 865.3 |
|  | Precision (CV) (%) | 11.7 | 8.9 | 1.6 | 1.4 |
|  | Accuracy (%) | -9.0 | -6.1 | 1.1 | 8.2 |
| Intra-day test batch 3  (n = 6) | Mean | 1.1 | 3.1 | 50.0 | 867.8 |
|  | Precision (CV) (%) | 16.4 | 11.4 | 4.5 | 2.1 |
|  | Accuracy (%) | 14.8 | 3.7 | 4.2 | 8.5 |
| Inter-day test  (n = 18) | Mean | 1.1 | 2.9 | 48.4 | 835.2 |
|  | Precision (CV) (%) | 18.6 | 10.1 | 5.7 | 5.8 |
|  | Accuracy (%) | 5.0 | -1.7 | 0.9 | 4.4 |
| **ATV** | | | | | |
| Variable | | LLOQ | Low QC | Medium QC | High QC |
| Nominal Concentration (pg/mL) | | 1.0 | 3.0 | 48.0 | 800.0 |
| Intra-day test batch 1  (n = 6) | Mean | 1.1 | 3.1 | 44.6 | 685.2 |
|  | Precision (CV) (%) | 13.0 | 11.3 | 8.2 | 7.2 |
|  | Accuracy (%) | 10.8 | 4.1 | -7.1 | -14.4 |
| Intra-day test batch 2  (n = 6) | Mean | 1.1 | 3.2 | 50.6 | 850.5 |
|  | Precision (CV) (%) | 17.2 | 11.2 | 3.0 | 4.8 |
|  | Accuracy (%) | 9.2 | 8.1 | 5.4 | 6.3 |
| Intra-day test batch 3  (n = 6) | Mean | 1.1 | 3.2 | 49.7 | 855.7 |
|  | Precision (CV) (%) | 17.8 | 8.5 | 1.8 | 3.2 |
|  | Accuracy (%) | 6.8 | 5.3 | 3.6 | 7.0 |
| Inter-day test  (n = 18) | Mean | 1.1 | 3.2 | 48.2 | 797.1 |
|  | Precision (CV) (%) | 15.2 | 9.9 | 7.4 | 11.3 |
|  | Accuracy (%) | 8.9 | 5.8 | 0.3 | -0.4 |

**Supplementary Table S2** Stability tests of all five drugs

| **MDZ** | | | | |
| --- | --- | --- | --- | --- |
| Variable | | Low QC | Medium QC | High QC |
| Nominal Concentration (pg/mL) | | 1.5 | 20.0 | 200.0 |
| On-bench stability  (RT for 4 h)  (n = 6) | Mean | 1.5 | 18.5 | 183.5 |
|  | Recovery (%) | 100.3 | 92.4 | 91.7 |
|  | Precision (CV) (%) | 4.7 | 2.8 | 0.6 |
| On-machine stability  (2℃ - 8℃ for 24 h)  (n = 6) | Mean | 1.5 | 18.5 | 181.0 |
|  | Recovery (%) | 100.8 | 92.7 | 90.5 |
|  | Precision (CV) (%) | 7.6 | 5.6 | 2.7 |
| Freeze-thaw stability  (3 cycles)  (n = 6) | Mean | 1.4 | 19.3 | 190.8 |
|  | Recovery (%) | 94.7 | 96.7 | 95.4 |
|  | Precision (CV) (%) | 4.2 | 7.3 | 9.1 |
| **DAB** | | | | |
| Variable | | Low QC | Medium QC | High QC |
| Nominal Concentration (pg/mL) | | 30.0 | 400.0 | 4000.0 |
| On-bench stability  (RT for 4 h)  (n = 6) | Mean | 27.7 | 396.4 | 4134.7 |
|  | Recovery (%) | 92.2 | 99.1 | 103.4 |
|  | Precision (CV) (%) | 3.6 | 4.6 | 3.2 |
| On-machine stability  (2℃ - 8℃ for 24 h)  (n = 6) | Mean | 29.4 | 413.9 | 4349.7 |
|  | Recovery (%) | 98.1 | 103.5 | 108.7 |
|  | Precision (CV) (%) | 3.5 | 2.7 | 2.4 |
| Freeze-thaw stability  (3 cycles)  (n = 6) | Mean | 31.9 | 398.1 | 4223.1 |
|  | Recovery (%) | 106.3 | 99.5 | 105.6 |
|  | Precision (CV) (%) | 7.3 | 6.7 | 4.7 |
| **PTV** | | | | |
| Variable | | Low QC | Medium QC | High QC |
| Nominal Concentration (pg/mL) | | 3.0 | 48.0 | 800.0 |
| On-bench stability  (RT for 4 h)  (n = 6) | Mean | 2.9 | 52.8 | 824.7 |
|  | Recovery (%) | 97.7 | 110.0 | 103.1 |
|  | Precision (CV) (%) | 9.4 | 3.9 | 3.1 |
| On-machine stability  (2℃ - 8℃ for 24 h)  (n = 6) | Mean | 3.2 | 48.9 | 840.8 |
|  | Recovery (%) | 105.2 | 101.8 | 105.1 |
|  | Precision (CV) (%) | 3.1 | 3.0 | 1.3 |
| Freeze-thaw stability  (3 cycles)  (n = 6) | Mean | 3.3 | 50.0 | 882.5 |
|  | Recovery (%) | 110.3 | 104.1 | 110.3 |
|  | Precision (CV) (%) | 3.4 | 1.6 | 1.7 |
| **RSV** | | | | |
| Variable | | Low QC | Medium QC | High QC |
| Nominal Concentration (pg/mL) | | 3.0 | 48.0 | 800.0 |
| On-bench stability  (RT for 4 h)  (n = 6) | Mean | 2.8 | 51.8 | 840.1 |
|  | Recovery (%) | 93.5 | 108.0 | 105.0 |
|  | Precision (CV) (%) | 10.4 | 4.5 | 2.5 |
| On-machine stability  (2℃ - 8℃ for 24 h)  (n = 6) | Mean | 3.1 | 47.4 | 769.0 |
|  | Recovery (%) | 103.8 | 98.7 | 96.1 |
|  | Precision (CV) (%) | 8.5 | 4.7 | 5.8 |
| Freeze-thaw stability  (3 cycles)  (n = 6) | Mean | 2.9 | 50.1 | 874.6 |
|  | Recovery (%) | 97.0 | 104.4 | 109.3 |
|  | Precision (CV) (%) | 7.8 | 4.9 | 2.2 |
| **ATV** | | | | |
| Variable | | Low QC | Medium QC | High QC |
| Nominal Concentration (pg/mL) | | 3.0 | 48.0 | 800.0 |
| On-bench stability  (RT for 4 h)  (n = 6) | Mean | 3.0 | 54.2 | 887.7 |
|  | Recovery (%) | 99.7 | 112.9 | 111.0 |
|  | Precision (CV) (%) | 11.2 | 5.0 | 7.7 |
| On-machine stability  (2℃ - 8℃ for 24 h)  (n = 6) | Mean | 2.8 | 54.4 | 852.4 |
|  | Recovery (%) | 94.3 | 113.3 | 106.5 |
|  | Precision (CV) (%) | 6.3 | 1.1 | 6.4 |
| Freeze-thaw stability  (3 cycles)  (n = 6) | Mean | 3.1 | 52.3 | 839.4 |
|  | Recovery (%) | 103.7 | 109.0 | 104.9 |
|  | Precision (CV) (%) | 7.5 | 6.8 | 5.9 |

RT: room temperature

**Supplementary Table S3** *In vitro* plasma protein binding of each drug in plasma from HVs and ESRD patients

|  | % Unbound (mean ± SD) *^a^* | | | | |
| --- | --- | --- | --- | --- | --- |
| Population | PTV | DAB | RSV | MDZ | ATV |
| Healthy | 0.12±0.01 | 59.3±5.77 | 3.65±0.33 | 0.50±0.03 | 1.24±0.08 |
| ESRD | 0.18±0.03 | 55.9±3.34 | 9.48±0.82 | 0.81±0.03 | 1.92±0.16 |

*^a^* Mean and standard deviation (SD) obtained from 3 replicates.

**Supplementary Table S4** Calculated PK parameters of each drug for unbound plasma concentrations in two groups

| Drug | Group | HV | ESRD | ESRD/HV ratio | P value | ESRD/HV ratio  (Caucasian)^b^ |
| --- | --- | --- | --- | --- | --- | --- |
| PTV | C_max_  (pg/mL) | 0.266  (0.220-0.324)^a^ | 0.443  (0.342-0.574) | 1.66 | <0.05 | 2.45 |
|  | AUC_last_  (h·pg/mL) | 0.600  (0.527-0.683) | 1.66  (1.24-2.23) | 2.77 | <0.05 | 2.17 |
| DAB | C_max_  (pg/mL) | 180  (154-210) | 215  (177-261) | 1.19 | 0.16 | 0.69 |
|  | AUC_last_  (h·pg/mL) | 1324  (1124-1560) | 3841  (3115-4736) | 2.90 | <0.05 | 3.23 |
| RSV | C_max_  (pg/mL) | 2.67  (1.92-3.69) | 5.56  (4.41-7.01) | 2.08 | <0.05 | 1.30 |
|  | AUC_last_  (h·pg/mL) | 21.6  (16.1-29.0) | 62.2  (48.6-79.6) | 2.88 | <0.05 | 0.89 |
| MDZ | C_max_  (pg/mL) | 0.244  (0.175-0.340) | 0.134  (0.078-0.232) | 0.55 | 0.05 | 0.85 |
|  | AUC_last_  (h·pg/mL) | 0.605  (0.459-0.800) | 0.275  (0.169-0.449) | 0.46 | <0.05 | 0.62 |
| ATV | C_max_  (pg/mL) | 0.293  (0.217-0.397) | 0.422  (0.250-0.712) | 1.44 | 0.25 | 4.00 |
|  | AUC_last_  (h·pg/mL) | 1.75  (1.38-2.21) | 3.48  (2.15-5.61) | 1.99 | <0.05 | 1.54 |

^a^ The parameter is shown as geometry mean (95% CI).

**Supplementary** **Table** **S5** Gene polymorphisms in all subjects

| **DMET** | **SNP** | **Genotype** | **N (%)** |
| --- | --- | --- | --- |
| CYP3A4 | rs2246709 | AA | 6 (31.5) |
|  |  | GG | 5 (26.3) |
|  |  | GA | 8 (42.1) |
| CYP3A4 | rs28371759 | AA | 18 (94.7) |
|  |  | GA | 1 (5.26) |
| CYP3A4 | rs4646437 | AA | 1 (5.26) |
|  |  | GG | 13 (68.4) |
|  |  | GA | 5 (26.3) |
| CYP3A4 | rs4986913 | GG | 19 (100) |
| CYP3A4 | rs55901263 | GG | 19 (100) |
| CYP3A4 | rs2242480 | CC | 12 (52.2) |
|  |  | TT | 5 (21.7) |
|  |  | TC | 6 (26.1) |
| CYP3A4 | rs35599367 | GG | 19 (100) |
| CYP3A4 | rs3735451 | CC | 5 (22.7) |
|  |  | CT | 7 (31.8) |
|  |  | TT | 10 (45.5) |
| CYP3A4 | rs55951658 | TT | 19 (100) |
| CYP3A5 | rs776746 | TT | 4 (17.4) |
|  |  | TC | 9 (39.1) |
|  |  | CC | 10 (43.5) |
| P-gp | rs1045642 | AA | 1 (4.35) |
|  |  | AG | 13 (56.5) |
|  |  | GG | 9 (39.1) |
| P-gp | rs1128503 | AA | 6 (31.6) |
|  |  | GG | 6 (31.6) |
|  |  | GA | 7 (36.8) |
| P-gp | rs2032582 | AA | 3 (12.5) |
|  |  | AT | 3 (12.5) |
|  |  | CC | 6 (25.0) |
|  |  | CA | 8 (33.3) |
|  |  | CT | 2 (8.33) |
|  |  | TT | 2 (8.33) |
| BCRP | rs2231137 | CC | 11 (47.8) |
|  |  | TT | 2 (8.70) |
|  |  | TC | 10 (43.5) |
| BCRP | rs2231142 | GG | 10 (43.5) |
|  |  | GT | 13 (56.5) |
| OATP1B1 | rs2306283 | AA | 1 (4.17) |
|  |  | AG | 7 (29.2) |
|  |  | GG | 16 (66.7) |
| OATP1B1 | rs4149056 | CC | 2 (10.5) |
|  |  | TT | 15 (78.9) |
|  |  | TC | 2 (10.5) |
| OATP1B3 | rs7311358 | AA | 9 (47.4) |
|  |  | GA | 10 (52.6) |
| OATP1B3 | rs4149117 | G | 12 (52.2) |
|  |  | GT | 10 (43.5) |
|  |  | T | 1 (4.35) |

**Supplementary** **Table S6. Multiple linear regression analysis results in ESRD patients**

| Drug | Variables | Regression coefficient | Intercept | CV (%) |
| --- | --- | --- | --- | --- |
| PTV | *Phascolarctobacterium* | 0.0124 | 17.5 | 16.9 |
|  | Urea | -0.273 |  |  |
|  | *Veillonella* | -2.20 |  |  |
| DAB | Hematocrit | -46.4 | 44.1 | 12.6 |
|  | *Clostridium_XVIII* | -8.77 |  |  |
| RSV | Age | 1.51 | -55.9 | 39.0 |
|  | creatine kinase_MB | 5.16 |  |  |
| MDZ | Triglyceride | 11.6 | 57.4 | 19.7 |
|  | *Anaerostipes* | -17.7 |  |  |
|  | Platelet count | 0.259 |  |  |
| ATV | Height | 8.92 | -1152 | 27.4 |
|  | *Sutterella* | 18.6 |  |  |

**Supplementary Table S7** PK parameters of simulated concentration-time curve for each drug

| Parameter *^a^* | Group | PTV | DAB | RSV | MDZ | ATV |
| --- | --- | --- | --- | --- | --- | --- |
| C_max_ (pg/mL) | HV | 313 (0.44) | 301 (20.1) | 66.1 (37.9) | 44.1 (39.1) | 45.1 (42.1) |
|  | ESRD | 317 (0.59) | 379 (22.0) | 56.0 (17.8) | 33.4 (10.3) | 52.8 (31.3) |
| AUC_last_ (h·pg/mL) | HV | 636 (12.1) | 2533 (22.3) | 941 (47.5) | 145 (43.6) | 253 (34.1) |
|  | ESRD | 1106 (32.5) | 17385 (16.2) | 1151 (43.7) | 73.0 (30.3) | 281 (55.2) |
| AUC_inf_ (h·pg/mL) | HV | 636 (12.61) | 2533 (22.3) | 1184 (68.8) | 145 (43.6) | 255 (34.8) |
|  | ESRD | 1107 (32.8) | 19847 (6.41) | 1656 (85.3) | 73.0 (30.3) | 282 (55.7) |
| CL/F (L/h) | HV | 15.7 (12.6) | 148 (22.3) | 42.2 (68.8) | 69.1 (43.6) | 392 (34.8) |
|  | ESRD | 9.03 (3.8) | 18.9 (6.41) | 30.2 (85.3) | 137 (30.3) | 354 (55.7) |
| V_z_/F (L) | HV | 236 (7.38) | 1670 (43.5) | 5466 (27.7) | 1088 (25.7) | 9657 (37.7) |
|  | ESRD | 178 (12.4) | 1271 (44.2) | 4923 (22.4) | 1721 (21.7) | 5005 (64.9) |
| T_1/2_ (h) | HV | 10.4 (5.20) | 7.82 (44.6) | 89.8 (37.6) | 10.9 (16.8) | 17.1 (48.4) |
|  | ESRD | 13.6 (19.9) | 46.6 (38.8) | 113 (57.3) | 8.7 (8.33) | 9.78 (57.1) |

*^a^* Results are presented as geometric mean (CV%) or median (range) unless stated otherwise.

**Supplementary Table S8** Comparison of observed and predicted plasma concentration at 28 h in ESRD participants

| Concentration *^a^* | PTV | DAB | RSV | MDZ | ATV |
| --- | --- | --- | --- | --- | --- |
| Observed | 6.77±2.56 | 87.3±28.5 | 6.44±4.51 | 0.15±0.00 | 3.95±2.49 |
| Predicted | 8.40±5.61 | 193±48.5 | 7.09±3.75 | 0.20±0.15 | 2.79±2.32 |

*^a^* Results are presented as mean and standard deviation (SD).
